# Design and Synthesis of Novel Hybrid 8-Hydroxy Quinoline-Indole Derivatives as Inhibitors of Aβ Self-Aggregation and Metal Chelation-Induced Aβ Aggregation

**DOI:** 10.3390/molecules25163610

**Published:** 2020-08-08

**Authors:** Suresh K. Bowroju, Nirjal Mainali, Srinivas Ayyadevara, Narsimha R. Penthala, Sesha Krishnamachari, Samuel Kakraba, Robert J. Shmookler Reis, Peter A. Crooks

**Affiliations:** 1 Department of Pharmaceutical Sciences, College of Pharmacy, University of Arkansas for Medical Sciences, Little Rock, AR 72205, USA; skbowroju@uams.edu (S.K.B.); NRPenthala@uams.edu (N.R.P.); 2 Bioinformatics Program, University of Arkansas at Little Rock and University of Arkansas for Medical Sciences, Little Rock, AR 72205, USA; nmainali@ualr.edu (N.M.); SKAKRABA@uams.edu (S.K.); 3 Central Arkansas Veterans Healthcare Service, University of Arkansas for Medical Sciences, Little Rock, AR 72205, USA; sesha@uams.edu (S.K.); AyyadevaraSrinivas@uams.edu (S.A.); 4 Department of Geriatrics, College of Medicine, University of Arkansas for Medical Sciences, Little Rock, AR 72205, USA; ReisRobertJ@uams.edu

**Keywords:** Alzheimer’s disease, clioquinol analogues, hybrid 8-hydroxyquinoline-indole analogs, Aβ-aggregation, metal chelating agents

## Abstract

A series of novel hybrid 8-hydroxyquinoline-indole derivatives (**7a–7e**, **12a–12b** and **18a–18h**) were synthesized and screened for inhibitory activity against self-induced and metal-ion induced Aβ_1–42_ aggregation as potential treatments for Alzheimer’s disease (AD). In vitro studies identified the most inhibitory compounds against self-induced Aβ_1–42_ aggregation as **18c**, **18d** and **18f** (EC_50_ = 1.72, 1.48 and 1.08 µM, respectively) compared to the known anti-amyloid drug, clioquinol (**1**, EC_50_ = 9.95 µM). The fluorescence of thioflavin T-stained amyloid formed by Aβ_1–42_ aggregation in the presence of Cu^2+^ or Zn^2+^ ions was also dramatically decreased by treatment with **18c**, **18d** and **18f**. The most potent hybrid compound **18f** afforded 82.3% and 88.3% inhibition, respectively, against Cu^2+^- induced and Zn^2+^- induced Aβ_1–42_ aggregation. Compounds **18c**, **18d** and **18f** were shown to be effective in reducing protein aggregation in HEK-tau and SY5Y-APP_Sw_ cells. Molecular docking studies with the most active compounds performed against Aβ_1–42_ peptide indicated that the potent inhibitory activity of **18d** and **18f** were predicted to be due to hydrogen bonding interactions, π–π stacking interactions and π–cation interactions with Aβ_1–42,_ which may inhibit both self-aggregation as well as metal ion binding to Aβ_1–42_ to favor the inhibition of Aβ_1–42_ aggregation.

## 1. Introduction

Alzheimer’s disease (AD) is the most debilitating age-associated neurodegenerative disorder leading to dementia, affecting millions of elderly people, and the number of patients is expected to reach 130 million worldwide by 2050 [1,2]. AD remains incurable due to the low efficacy and the very limited number of available drugs to treat this neurodegenerative disease. Salient features of AD are the accumulation of amyloid-β (Aβ) plaques and tangles containing hyperphosphorylated tau protein. These proteins misfold and aggregate in the brains of affected individuals as amyloid senile plaques outside the neurons and neurofibrillary tangles (NFTs) within neurons, which are diagnostic for AD but accompanied by dyshomeostasis of biometals [3,4]. Current treatments for AD include acetylcholinesterase (AChE) inhibitors (i.e., tacrine, donepezil, rivastigmine, and galantamine), and *N*-methyl-D-aspartate (NMDA) antagonists, the only drugs approved for AD which provide a symptomatic relief strategy for mild forms of AD [5]. Unfortunately, there is currently no means to cure or even slow the progression of AD [6], spurring increased efforts to develop more effective drugs to prevent or treat AD. Due to the complexity of AD and the multitude of factors potentially involved in its progression, a strategy that uses multi-target directed ligands (MTDLs) has drawn much attention as a mainstream therapeutic approach for treatment of this disease [7,8,9,10,11].

The deposition of Aβ plays a crucial role in the pathogenesis of AD [12]. Many studies have shown that Aβ forms consist of several different types of aggregates, such as oligomers and fibrils [13,14]. Among these aggregates, soluble Aβ oligomers produce the most potent neurotoxicity and are generally regarded as the main neurotoxins in AD. Soluble Aβ oligomers not only lead to cognitive impairment in rodents but also induce neuronal death in primary neurons and neuronal cell cultures [15]. Soluble Aβ oligomers rapidly interact with neuronal cell membranes, produce free radicals, and increase the levels of intracellular reactive oxygen species (ROS) [16]. Moreover, Aβ oligomers induce neuronal apoptosis by regulating signaling pathways, such as glycogen synthase kinase 3β (GSK3β) and mitogen-activated protein kinase (MEK)/extracellular signal-regulated kinase (ERK) signaling [16]. In addition, metal ions such as Cu^2+^, Zn^2+^, Fe^2+^, and Fe^3+^ are required for neuronal activity within synapses. Due to the necessity of these metal ions, cells have a sophisticated system to maintain metal-ion homeostasis. Breakdown of these mechanisms alters the ionic balance and can result in aggregation of Aβ peptide into plaques, and production of reactive oxygen species induced by Aβ. Elevated levels of these metal ions can readily bind to Aβ via histidine residues H6, H13, and H14, facilitating Aβ aggregation [17,18,19,20,21] and generating reactive oxygen species (ROS) via Fenton-like reactions, which lead to oxidative stress and eventual neuronal death in AD patients [22]. Thus, clearance of Aβ amyloid in the brain by targeting metal ions effectively detoxifies the Aβ plaques, and has become a promising approach for inhibition of Aβ aggregation [17,20,22]. These metal ions are also implicated in the formation of hyperphosphorylated tau and tau tangles [23].

**Figure 1:**
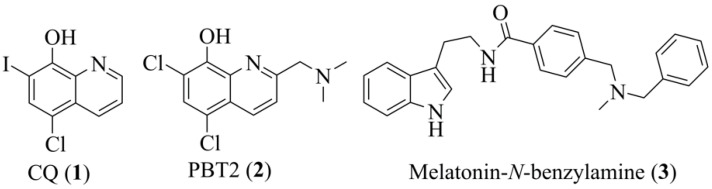
Chemical structures of multitarget-directed drugs clioquinol (**1**), PBT2 (**2**), and melatonin-*N*-benzylamine (**3**).

**Figure 2:**
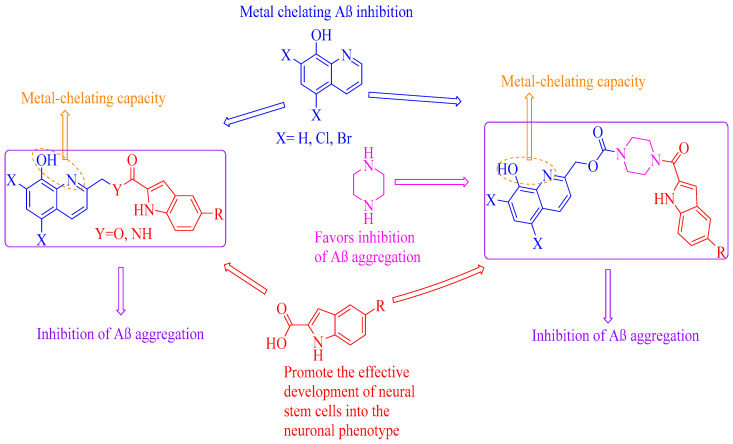
Design strategy for hybrid 8-hydroxyquinoline-indole ester and amide analogs with and without a piperazine-bridging moiety as multitarget-directed drugs for Alzheimer’s disease (AD) therapy.

Clioquinol (CQ, **1**, Figure 1), and PBT2 (**2**,**Figure 1**) exhibit moderate affinity for Cu^2+^ and Zn^2+^ and can inhibit metal-induced Aβ aggregation and ROS generation in vitro [24,25,26,27]. Recently, 8-hydroxyquinoline-based multi-target-directed ligands (MTDLs), such as M30, HLA20, WBQ5187, and tacrine-8-hydroxyquinoline hybrids, have been developed for treatment of AD. These compounds significantly inhibit Aβ aggregation in vitro and improve cognition in vivo in mouse models [28,29,30,31]. Interestingly, the melatonin-*N*-benzylamine hybrids (**3**, Figure 1), which have an indole framework, can promote the effective development of neural stem cells into the neuronal phenotype [32,33]. The current study focuses on a series of molecules that incorporate the indole moiety and the 8-hydroxyquinoline moiety into a novel 8-hydroxyquinoline-indole hybrid structure (Figure 2; left panel). The fifth and seventh position of the 8-hydroxyquinoline moiety has also been varied by replacing the hydrogen atoms with chloro or bromo groups, followed by conjugation of these 8-hydroxyquinoline moieties to indole or 5 fluoro-, 5-chloro- or 5-methoxyindole scaffolds to improve binding affinity and metal ion selectivity. In addition, recent studies have reported that incorporating a piperazine moiety into these MTDL molecules affords significant inhibition of Aβ aggregation [34], which prompted us to design a second set of hybrid molecules by inserting a piperazine bridging moiety between the 8-hydroxyquinoline and indole scaffolds (Figure 2; right panel) to afford a series of novel 8-hydroxyquinoline indole ester and amide analogs as multitarget-directed drugs in AD.

Taking into account neuronal loss, deficits in adult neurogenesis, and the formation of plaques and tangles associated with AD, the present work is focused on the identification of pharmacophores containing the 8-hydroxyquinoline moiety conjugated to an indole scaffold, in order to generate a series of hybrid 8-hydroxyquinoline indole esters and amides without (**7a–7e** and **12a–12b**) and with a piperazine bridging moiety (**18a–18h**). Fifteen novel hybrid 8-hydroxyquinoline-indole analogs of this type were prepared and investigated for their inhibition of Aβ_1–42_ self-aggregation and metal ion-induced aggregation. Compounds which showed inhibitory activity against self-induced Aβ_1–42_ aggregation were also evaluated against HEK-tau and SY5Y-APP_Sw_ cells to determine their effects on cellular protein aggregation.

**Scheme 1:**
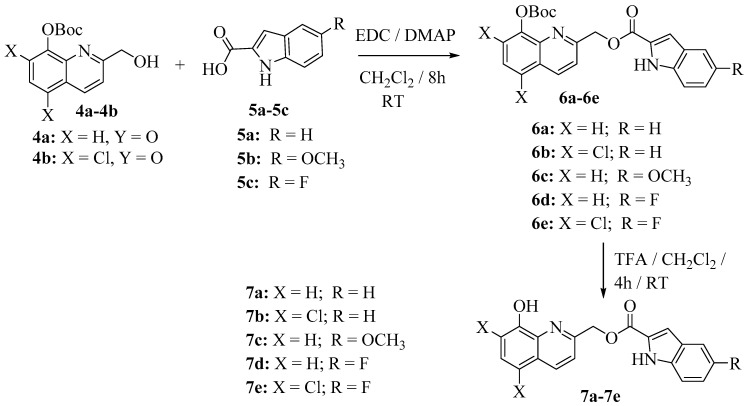
Synthesis of (8-hydroxyquinolin-2-yl)methyl 1*H*-indole-2-carboxylate analogs **7a–7e.**

For the synthesis of hybrid 8-hydroxyquinoline indoles (**7a–7e**), we initially prepared *tert*-butyl (2-(hydroxymethyl)quinolin-8-yl) carbonates **4a** and **4b** utilizing literature procedures [35], and carried out EDC coupling of each compound with a variety of indole-2-carboxylic acids (**5a–5c**) to afford Boc-protected ester intermediates **6a–6e**. Boc-deprotection of intermediates **6a–6e** was carried out with trifluoroacetic acid in dichloromethane at room temperature to afford **7a–7e** (Scheme 1).

**Scheme 2:**
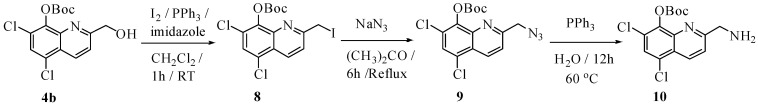
Synthesis of 2-(aminomethyl)-5,7-dichloroquinolin-8-yl *tert*-butyl carbonate intermediate **10.**

For the synthesis of hybrid *N*-((5,7-dichloro-8-hydroxyquinolin-2-yl)methyl)-1*H*-indole-2-carboxamides (**12a–12b**), the key intermediate 2-(aminomethyl)-5,7-dichloroquinolin-8-yl *tert*-butyl carbonate (**10**) was prepared by reacting intermediate **4b** [35] with iodine in the presence of PPh_3_ and imidazole in dichloromethane, for 1 h at room temperature, to afford iodo-intermediate **8**. The product was then reacted with sodium azide, at reflux temperature in acetone for 6 h, to afford the azido intermediate **9**. Compound **9** was then reduced to the amino compound **10** by treatment with PPh_3_ in water at 60 °C for 12 h (Scheme 2).

**Scheme 3:**
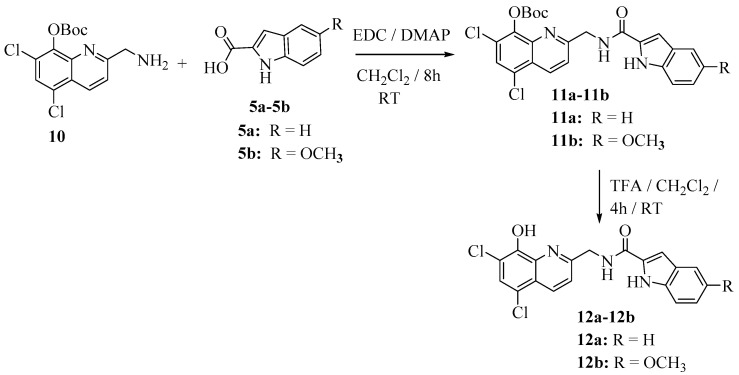
Synthesis of *N*-((5,7-dichloro-8-hydroxyquinolin-2-yl)methyl)-1*H*-indole-2-carboxamide analogs (**12a** and **12b**).

Finally, EDC coupling of **10** with indole-2-carboxylic acids **5a** or **5b** afforded the Boc-protected amide intermediates **11a** and **11b.** Boc-deprotection of **11a** and **11b** was carried out with trifluoroacetic acid in dichloromethane at room temperature to afford **12a** and **12b**, respectively (Scheme 3).

**Scheme 4:**
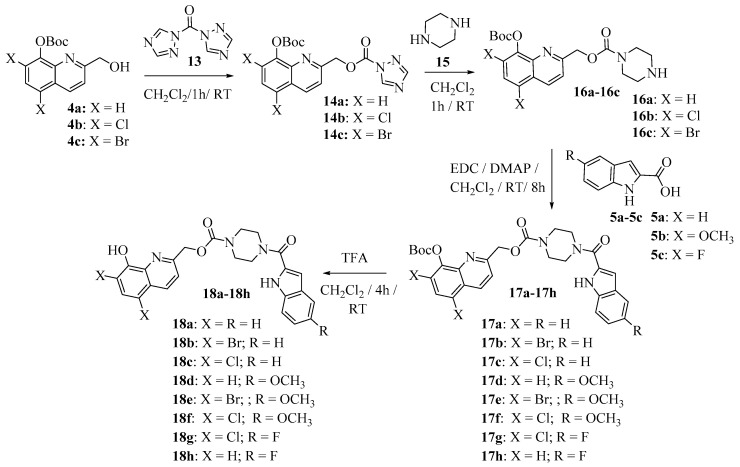
Synthesis of (8-hydroxyquinolin-2-yl)methyl-4-(1*H*-indole-2-carbonyl)piperazine-1- carboxylate analogs **18a–18h.**

For the synthesis of hybrid (8-hydroxyquinolin-2-yl)methyl-4-(1*H*-indole-2-carbonyl)piperazine-1-carboxylates **18a–18h**, initially we synthesized 8-(*tert*-butoxycarbonyloxyquinolin-2-yl)methyl 1*H*-1,2,4-triazole-1- carboxylate intermediates **14a–14c** by reacting *tert*-butyl (2-(hydroxymethyl)quinolin-8-yl) carbonates **4a–4c** with di-(1*H*-1,2,4-triazol-1-yl)methanone (**13**, CDT) in dichloromethane at room temperature. Compounds **14a–14c** were then each reacted with piperazine (**15**) in dichloromethane at room temperature to afford the corresponding piperazine-1-carboxylates **16a–16c**, which were subjected to EDC coupling with indole-2-carboxylic acids **5a–5c** to afford the Boc-protected intermediates **17a–17h**. Subsequent deprotection of **17a–17h** with trifluoroacetic acid in dichloromethane at room temperature over 4 h afforded the desired series of (8-hydroxyquinolin-2-yl)methyl-4-(1*H*-indole-2-carbonyl)piperazine-1-carboxylates **18a–18h** in 75**–**90% yields (Scheme 4). Structural confirmation of all the above intermediates and final synthetic products was obtained from ^1^H-NMR, ^13^C-NMR, and HR-MS analysis (See Supplementary Materials).

## 2. Results and Discussion

**Figure 3:**
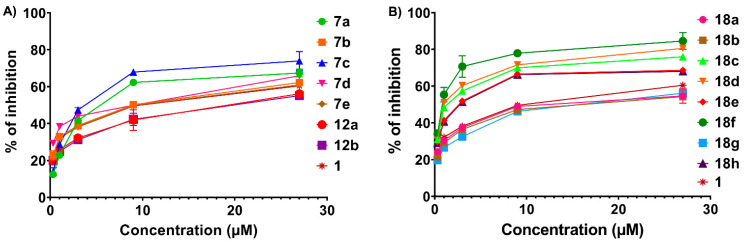
Dose–response curves of (**A**) compounds **7a**–**7e, 12a**–**12b** and **1;** (**B**) compounds **18a**–**18h** and **1** against self-induced Aβ_1–42_ aggregation.

The novel hybrid 8-hydroxyquinoline-indole derivatives, **7a–7e**, **12a, 12b**, and the (8-hydroxyquinolin-2-yl)methyl-4-(1*H*-indole-2-carbonyl)piperazine-1-carboxylate analogs **18a–18h** were evaluated for inhibitory activity against self-induced Aβ_1–42_ aggregation at five different concentrations (0.3, 1.0, 3, 9 and 27 µM) with 25 µM Aβ_1–42_, utilizing a thioflavin T (ThT) fluorescence assay with clioquinol, 1 as a positive control (Figure 3). In the absence of inhibitor, the self-induced Aβ_1–42_ aggregation ThT fluorescence was recorded as 115 arbitrary units (a.u.). The self-induced Aβ_1–42_ aggregation results for all the above synthetic analogs from the ThT fluorescence assay are shown in Table 1. The results indicate that analogs lacking a piperazino bridging moiety in their structure (i.e., **7a–7e** and **12a–12b**) were found to be either weak inhibitors or inactive against self-induced Aβ_1–42_ aggregation. Of the piperazino-containing compounds **18a–18h**, five analogs exhibited EC_50_ values >3 μM. 8-Hydroxyquinolin-2-yl compounds containing the piperazine bridge linked to indole (**18c**), or 5-methoxy indole (**18d** and **18f**) (EC_50_ = 1.72, 1.48 and 1.08 µM, respectively) were the most potent inhibitors of self-induced Aβ_1–42_ and were superior to both clioquinol (1, EC_50_ = 9.95 µM) and 1*H*-indole-2-carboxylic acid (5a, EC_50_ > 20 µM). The structure activity study (SAR) revealed that incorporation of the piperazino bridging moiety between a 5-methoxy indole group and a 5,7-dichloro-8-hydroxyquinoline group affords the most potent compound, **18f**, which exhibited a 10-fold improvement in inhibitory potency over **1**.

Analogs **18c**, **18d**, and **18f**, showed potent self-induced Aβ_1–42_ aggregation (<2 µM) and were selected for further investigation for inhibition of Cu^2+^- and Zn^2+^-induced Aβ_1–42_ aggregation. Metal ions such as Cu^2+^ and Zn^2+^ can react with Aβ to form Aβ oligomers, and then eventually to form Aβ amyloid plaques [8,14]. Due to their inhibitory activity against Aβ_1–42_, **18c**, **18d**, and **18f** were expected to decrease metal ion-induced Aβ_1–42_ aggregation.

**Figure 4:**
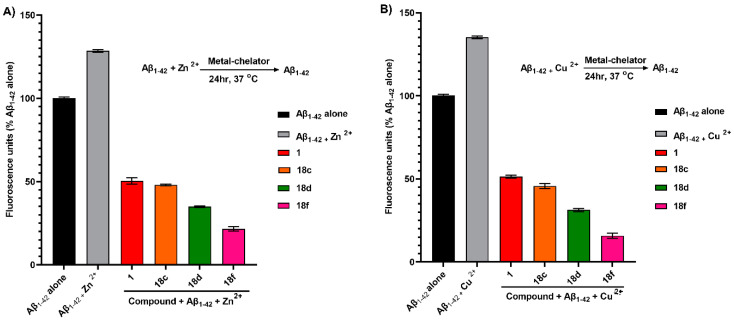
Effect of (**A**) Zn^2+^- induced and (**B**) Cu^2+^-induced Aβ_1–42_ aggregation in the presence clioquinol (**1**), **18c**, **18d**, and **18f** at 50 µM. Data shown are presented as the mean ±SD of three independent experiments.

Initially, CuSO_4_ or ZnCl_2_ was added to Aβ_1–42_ peptides at room temperature and the mixture incubated with or without drugs for 24 h at 37 °C. The fluorescence intensity of Aβ_1–42_ peptide alone was designated as 100%. The fluorescence intensity of Aβ_1–42_ peptide after treatment with Cu^2+^ was 135.1% and after treatment with Zn^2+^ was 129.3% compared to Aβ_1–42_ alone (Figure 4A,B), indicating that both Cu^2+^ and Zn^2+^ ions significantly enhanced Aβ_1–42_ aggregation.

In contrast, the fluorescence produced by Aβ_1–42_ treated with Cu^2+^ or Zn^2+^ ions in the presence of **18c, 18d**, and **18f** dramatically decreased (Figure 4), indicating inhibition of metal ion-induced Aβ_1–42_ aggregation (i.e., **18c**, 76.6% decrease (Cu^2+^) and 70.3% decrease (Zn^2+^); **18d**, 66.1% decrease (Cu^2+^) and 59.3% decrease (Zn^2+^); **18f**, 88.3% decrease (Cu^2+^) and 82.3% decrease (Zn^2+^); and for comparison compound **1**, 62.5% decrease (Cu^2+^) and 57.2% decrease (Zn^2+^)). These results show that **18f** exhibited significantly greater inhibitory activity in Cu^2+^- and Zn^2+^-induced Aβ_1–42_ aggregation than compound **1**.

**Figure 5:**
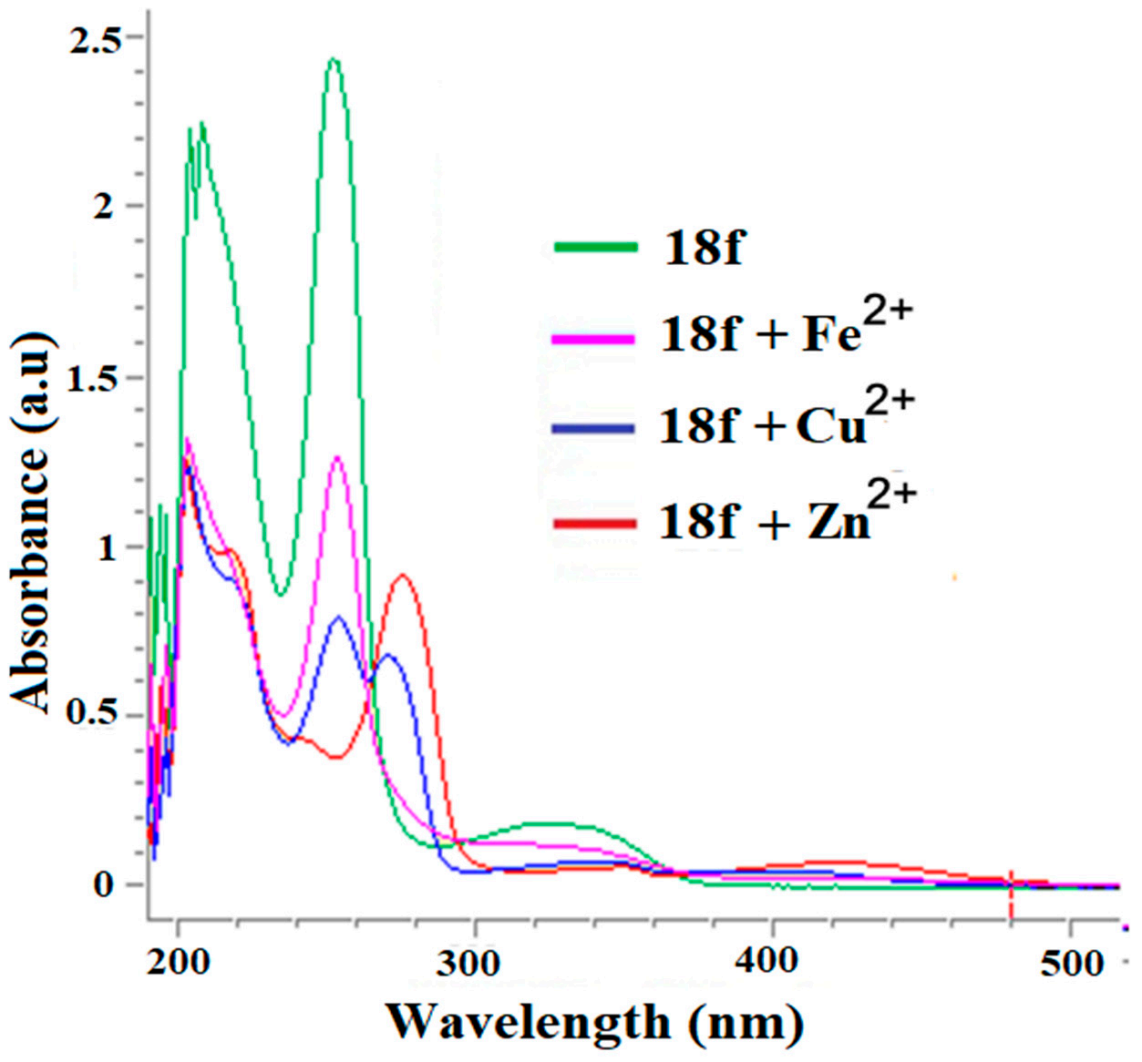
UV-absorbance spectra of compound **18f** (50 µM) in the presence of Cu^2+^, Zn^2+^, or Fe^2+^ (each at 50 µM) in buffer (20 mM HEPES, 150 mM NaCl; pH = 7.4). **18f** displayed maximum absorption wavelength shifts from 252 to 274, 269 and 255 nm in the presence of Cu^2+^, Zn^2+^, and Fe^2+^, respectively.

**Figure 6:**
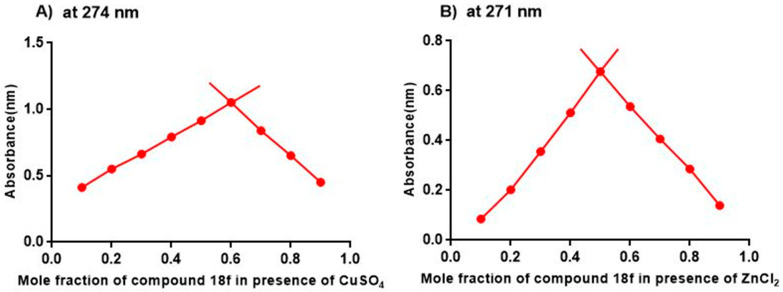
Determination of the stoichiometry of (**A**) **18f**-Cu^2+^ complex and (**B**) the **18f**-Zn^2+^ complex by Job’s method [34].

Based on the above results from self-induced Aβ_1–42_ aggregation and metal-induced Aβ_1–42_ aggregation studies, the most potent compounds **18f** was selected for further investigation into its ability to chelate metal ions such as Cu^2+^, Zn^2+^, and Fe^2+^ using UV-vis spectroscopy at wavelengths ranging from 200 to 500 nm. Compound **18f** (50 μM) was treated with 50 μM concentrations of CuSO_4_, ZnCl_2_, or FeSO_4_ for 30 min in HEPES buffer at room temperature, and displayed maximum absorption wavelength shifts from 252 to 274, 269 and 255 nm in the presence of Cu^2+^, Zn^2+^, and Fe^2+^, respectively (Figure 5). These wavelength shifts are indicative of the formation of **18f**-Cu^2+^, **18f**-Zn^2+^, and **18f**-Fe^+2^ and show that Zn^2+^ and Cu^2+^ undergo significant chelation with compound **18f**. The spectrum of **18f**-Fe^2+^ showed only a moderate increase in wavelength. Furthermore, the stoichiometry of formation of metal complexes **18f**-Cu^2+^ and **18f**-Zn^2+^ was also determined by using Job’s method (Figure 6) [36]. The isosbestic points shown in Figure 6A,B at 0.6 and 0.5 imply a stoichiometry of 1.5:1 for **18f**-Cu^2+^ and 1:1 for **18f**-Zn^2+^.

Copper and zinc dysregulation in the brain is considered to arise as a consequence of age-associated neurodegenerative disease such as Alzheimer’s disease. Zinc has also been shown to play an important role in pre- and post-synaptic responses [37]. Our drug design approach is to target dysregulation of metal homeostasis and protein aggregation. Dysregulation of metal ions is implicated in the elevated formation of reactive oxygen species and in susceptibility to oxidative stress. These metal ions were shown to interact with protein aggregation seed proteins such as tau and Aβ_1–42_, increasing misfolding and promoting protein aggregation. The 8-hydroxyquinoline compound, PBT2, has been shown to influence trans-synaptic effects of copper and zinc ions in a cell culture model and has been considered as a treatment for AD [38]. PBT2 (**2**) was also shown to induce amyloid degradation by inhibiting glycogen synthase kinase-3 activity via phosphorylation [38].

**Figure 7:**
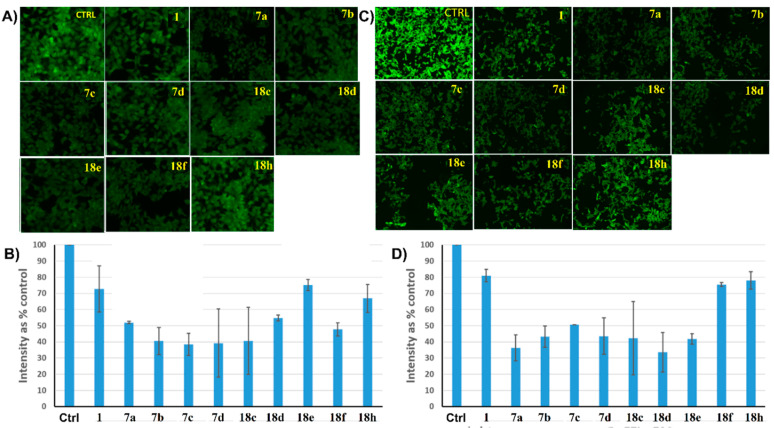
Amyloid-like aggregate intensity reduction by several analogs of hybrid 8-hydroxy quinoline-indoles in HEK-tau cells (**A**,**B**) and in SY5Y APP_Sw_ (neuroblastoma) cells (**C**,**D**).

In our study, we have shown that restoring metal homeostasis by treatment with hybrid 8-hydroxyquinoline-indole analogs reduces tau- and amyloid-mediated protein aggregation, and their associated neurotoxicity. Analogs **7a–7d, 18c–18f**, and **18h** were evaluated for their ability to reduce protein aggregation in HEK-tau cells. HEK-tau cells were exposed to the above Aβ_1__–__42_ aggregation inhibitors and parent drug clioquinol (**1**) at 1 µM for 48 h at 37 °C. Cells were assessed for amyloid deposits by staining with 0.1% *w/v* thioflavin T, counterstaining nuclei with DAPI, and mean ThT fluorescence signal per nucleus was calculated over multiple fields [39]. All the analogs reduced fluorescence intensity (indicative of protein aggregation) by 26**–**62% (*P* < 0.001 by 2-tailed *t*-test). Among these analogs, compounds **7b–7d, 18c**, and **18f** were the most effective as protein aggregation inhibitors in HEK-tau cells, and were more potent than clioquinol (**1**) (Figure 7A,B).

In another study compounds **7a–7d, 18c–18f**, and **18h** were also evaluated for their ability to reduce protein aggregation in SY5Y-APP_Sw_ cells. SY5Y-APP_Sw_ cells were exposed to the above protein aggregation inhibitors and parent compound **1** at 1 µM for 48 h at 37 °C. Cells were assessed for amyloid deposits by staining with 0.1% *w/v* ThT, counterstaining nuclei with DAPI, and mean ThT fluorescence signal per nucleus calculated over multiple fields. All the analogs reduced fluorescence intensity (indicative of protein aggregation) by 22–67% (*P* < 0.001 by 2-tailed *t*-test). Analogs **7a** and **18d** were the most effective in SY5Y-APP_Sw_ cells and were more potent than the parent compound, clioquinol (**1**) (Figure 7C,D). Thioflavin-T fluorescence assays for in vitro amyloid aggregation and cell culture models of amyloid aggregation overlap but are not identical. This is due to the fact that thioflavin in cell-culture stains not just amyloid aggregates but also other amyloid-like fibrillar structures. Another important difference is that in vitro aggregation is assessed after 24 h, whereas aggregation in cell culture is assessed after 48 h.

**Figure 8:**
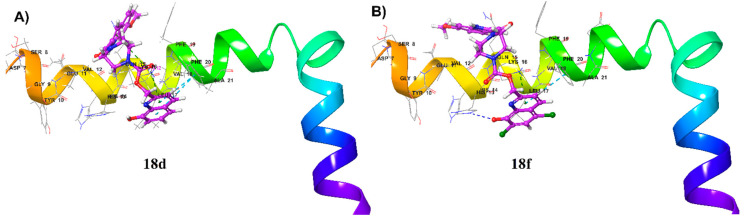
Proposed binding modes of (**A**) compound **18d** and (**B**) **18f** with Aβ_1–42_ peptide.

The binding interactions of the active compounds **18d** and **18f** were also investigated utilizing a crystal structure of the target Aβ_1–42_ peptide (PDB code: 1IYT) [40] employing Schrodinger Maestro 11.4 software (Figure 8) [41]. For analogue **18d**, the quinoline ester carbonyl group of this hybrid molecule is involved in a hydrogen bonding interaction with Lys16 at a distance of 3.12 A^o^ units and the 8-hydroxyquinoline moiety is involved in a π–π stacking interaction with residue Phe20; this is similar to that observed with the deoxyvasicinone-donepezil hybrid molecule binding region on Aβ_1–42_ reported in the literature [41]. In addition, the quinolone and indole ring systems of compound **18d** are involved in π–cation interactions with Lys16. With the potent analog **18f**, the hydroxyl group of the 8-hydroxyquinoline moiety is involved in a hydrogen bonding interaction with the His13 residue at 2.17 A^o^ units, and the 8-hydroxyquinoline moiety of **18f** is also involved in a π–π stacking interaction with Phe20, similar to the binding mode of compound **18d**. In addition, the quinoline ring system of **18f** is involved in a π–cation interaction with Lys16. These docking results indicate that the effective inhibition of both self-induced and metal ion-induced Aβ_1–42_ aggregation by **18d** and **18f** may be due to hydrogen bonding interactions, π–π stacking interactions, and π–cation interactions of these molecules. Of particular interest is the interaction the most potent compound **18f** with the His13 residue on the Aβ_1–42_ peptide. His13 has been implicated in the binding of metal ions with Aβ_1–42_ (17–21); thus, **18f** may compete with metal ions for this binding site on Aβ_1–42_, thereby inhibiting metal ion induction of Aβ_1–42_ aggregation.

In summary, a series of hybrid 8-hydroxyquinoline-indole derivatives **7a–7e, 12a–12b**, and **18a–18h** have been synthesized and evaluated as inhibitors of Aβ_1–42_ self-aggregation and metal chelation-induced aggregation, and have also been evaluated against HEK-tau and SY5Y-APP_Sw_ cells to determine their effect on cellular protein aggregation. In vitro studies showed that hybrid 8-hydroxyquinoline-indole analogs lacking the piperazine bridging unit (i.e., compounds **7a–7e** and **12a–12b**) exhibited poor-to-moderate inhibitory activities against Aβ_1–42_ peptide self-aggregation. However, the majority of the hybrid 8-hydroxyquinoline-indole compounds incorporating the piperazine bridge unit (i.e., **18c–18f**, and **18h)** were potent inhibitors against Aβ_1–42_ self-aggregation_._ Analogue **18f** was the most potent inhibitor of Aβ_1–42_ aggregation in both self-induced and metal chelation-induced assays, and in protein aggregation assays in HEK-tau cells. Compound **18f** afforded 82.3% and 88.3% inhibition against Cu^2+^-induced and Zn^2+^-induced Aβ_1–42_ aggregation assays, and was also shown to be a chelator of Cu^2+^, Zn^2+^, and Fe^2+^ ions in physiological buffer solutions. Molecular docking studies with **18f** showed that this molecule has the potential to interact with the His13 residue on Aβ_1–42_ peptide, which suggests that its potent anti-aggregation properties may also be due in part to its ability to competitively inhibit the binding of metal ions that interact with this site to induce Aβ_1–42_ aggregation. Thus, the present study has identified **18f,** an analog that incorporates a piperazine bridging group between a 5-methoxyindole moiety and a 5,7-dichloro-8-hydroxyquinoline moiety, as the most potent Aβ_1-42_ aggregation inhibitor; this analog exhibited a 10-fold improvement in inhibitory potency over clioquinol (**1**) as an inhibitor of Aβ_1–42_ self-aggregation. We consider **18f** to be a lead compound worthy of further structural optimization and preclinical development as a treatment for AD.

## 3. Experimental Section

### 3.1. Chemistry

All reagents, solvents, and chemicals utilized in the synthesis of the hybrid 8-hydroxyquinoline-indole analogs were purchased from Oakwood Chemicals Fisher Scientific and Adooq Bioscience. ^1^H and ^13^C-NMR spectra were recorded on a Varian 400 MHz spectrometer equipped with a Linux workstation running on vNMRj software. Spectral analyses were carried out in CDCl_3_ or DMSO-*d*_6_ for both ^1^H and ^13^C spectra. Chemical shifts were measured in δ parts per million (*ppm*) and coupling constants (*J*) were measured in hertz (Hz). High-resolution mass spectra (HR-MS) were recorded on an Agilent 6545 ESI/APCI TOF MS. Thin-layer chromatography (TLC) was carried out on pre-coated silica gel glass plates (F254 Merck).

### 3.2. Experimental Procedure for the Synthesis of Hybrid 8-Hydroxyquinoline-Indole Analogs

Synthesis of (8-hydroxyquinolin-2-yl)methyl-*1H*-indole-2-carboxylate hybrid analogs **7a–7e**

To a stirred solution of an appropriate indole-2-carboxylic acid (**5a–5c**) (0.15 mmol) in dry dichloromethane (5 mL) under an argon atmosphere at 0 °C was added 1-ethyl-3-(3-dimethylamino propyl)carbodiimide (EDCI, 0.181 mmol), and the mixture allowed to stir for 15 min. A catalytic amount of 4-dimethylaminopyridine (DMAP, 0.045 mmol) was then added, followed by addition of an appropriate *tert*-butyl (2-(hydroxymethyl)quinolin-8-yl) carbonate **(4a–4b)** (0.151 mmol) in dry dichloromethane (3 mL) at 0 °C. The resulting mixture was stirred for 8 h at room temperature. After completion of the reaction (monitored by TLC), the reaction mixture was washed with water (10 mL), and the dichloromethane layer separated, dried over anhydrous sodium sulfate, filtered, concentrated, and purified by silica gel column chromatography using 2**–**5% methanolic dichloromethane as eluent to afford the corresponding Boc-protected 8-hydroxyquinoline-indole derivatives (**6a–6e**).

The above Boc-protected 8-hydroxyquinoline-indole derivatives **(6a–6e)** were each dissolved in a mixture of DCM (10 mL) and TFA (1.0 mL) and stirred for 6 h until the Boc-deprotection was completed. A saturated NaHCO_3_ solution (20 mL) was then added and the resulting mixture was extracted with dichloromethane. The organic layer was separated and washed with water, followed by brine solution, dried over anhydrous Na_2_SO_4_, and concentrated to afford the appropriate hybrid 8-hydroxyquinoline indole derivatives (**7a–7e**).

*(8-Hydroxyquinolin-2-yl)methyl-1H-indole-2-carboxylate (***7a***): *^1^H-NMR (400 MHz, DMSO-*d*_6_): δ 12.00 (s, 1H), 9.67 (s, 1H), 8.38 (d, *J* = 8.4 Hz, 1H), 7.76**–**63 (m, 2H), 7.52**–**7.37 (m, 3H), 7.35**–**7.22 (m, 2H), 7.13–7.07 (m, 2H), 5.64 (s, 2H) *ppm*. ^13^C-NMR (100 MHz, DMSO-*d*_6_): δ 161.44, 154.70, 153.40, 138.08, 138.01, 137.56, 128.57, 127.99, 127.16, 127.14, 125.34, 122.59, 120.73, 120.35, 118.15, 113.06, 112.19, 108.99, 67.50 *ppm*. HR-MS (ESI) *m*/*z* calcd for C_19_H_14_N_2_O_3_ (M + H)^+^ 319.1075, found 319.1076.

*(5,7-Dichloro-8-hydroxyquinolin-2-yl)methyl-1H-indole-2-carboxylate (***7b***): *^1^H-NMR (400 MHz, DMSO-*d*_6_): δ 12.02 (s, 1H), 10.67 (s, 1H), 8.58 (d, *J* = 8.8 Hz, 1H), 7.91 (d, *J* = 8.8 Hz, 1H), 7.84 (s, 1H), 7.7 (d, *J* = 8.0 Hz, 1H), 7.49 (d, *J* = 8.8 Hz, 1H), 7.33 (s, 1H), 7.29 (t, *J* = 7.6 Hz, 1H), 7.10 (t, *J* = 7.6 Hz, 1H), 5.70 (s, 2H) *ppm*. ^13^C-NMR (100 MHz, DMSO-*d*_6_): δ 161.35, 156.98, 149.23, 138.68, 138.05, 134.50, 128.27, 127.15, 126.97, 125.42, 124.61, 122.62, 121.61, 120.77, 119.58, 116.56, 113.06, 109.14, 67.02 *ppm*. HR-MS (ESI) *m*/*z* calcd for C_19_H_12_Cl_2_N_2_O_3_ (M + H)^+^ 387.0291, found 387.0296.

*(8-Hydroxyquinolin-2-yl)methyl-5-methoxy-*1*H-indole-2-carboxylate (***7c***): *^1^H-NMR (400 MHz, DMSO-*d*_6_): δ 11.87 (s, 1H), 9.67 (s, 1H), 8.38 (d, *J* = 8.4 Hz, 1H), 7.70 (d, *J* = 8.4 Hz, 1H), 7.46–7.35 (m, 3H), 7.2 (s, 1H), 7.12 (d, *J* = 2.8 Hz, 2H), 6.95 (d, *J* = 8.8 Hz, 1H), 5.63 (s, 2H), 3.76 (s, 3H) *ppm*.^13^C-NMR (100 MHz, DMSO-*d*_6_): δ 161.36, 154.76, 154.44, 153.40, 138.08, 137.55, 133.40, 128.57, 127.98, 127.49, 127.30, 120.33, 118.15, 117.03, 113.98, 112.18, 108.55, 102.39, 67.40, 55.65 *ppm*. HR-MS (ESI) *m*/*z* calcd for C_20_H_16_N_2_O_4_ (M + H)^+^ 349.1174, found 349.1178.

*(8-Hydroxyquinolin-2-yl)methyl-5-fluoro-1H-indole-2-carboxylate (***7d***): *^1^H-NMR (400 MHz, DMSO-*d*_6_): δ 12.13 (s, 1H), 9.67 (s, 1H), 8.38 (d, *J* = 8.4 Hz, 1H), 7.71 (d, *J* = 8.4 Hz, 1H), 7.50–7.40 (m, 4H), 7.28 (s, 1H), 7.19–7.11 (m, 2H), 5.64 (s, 2H) *ppm*. ^13^C-NMR (100 MHz, DMSO-*d*_6_): δ 161.17, 158.86, 156.54, 154.58, 153.41, 138.09, 137.57, 134.75, 128.80, 128.58, 128.01, 127.24, 127.13, 120.37, 118.15, 114.49, 114.39, 114.20, 112.20, 108.88, 108.83, 106.66, 106.43, 67.62 *ppm*. HR-MS (ESI) *m*/*z* calcd for C_19_H_13_FN_2_O_3_ (M + H)^+^ 337.0969, found 337.0978.

*(5,7-Dichloro-8-hydroxyquinolin-2-yl)methyl-5-fluoro-1H-indole-2-carboxylate (***7e***): *^1^H-NMR (400 MHz, DMSO-*d*_6_): δ 12.14 (s, 1H), 10.64 (s, 1H), 8.57 (d, *J* = 8.8 Hz, 1H), 7.91 (d, *J* = 8.8 Hz, 1H), 7.83 (s, 1H), 7.50 (m, 2H), 7.30 (d, *J* = 2.0 Hz, 1H), 7.16 (td, *J* = 9.2, 2.4 Hz, 1H), 5.67 (s, 2H) *ppm*; ^13^C-NMR (100 MHz, DMSO-*d*_6_): δ 161.09, 158.87, 156.83, 156.54, 149.20, 138.66, 134.78, 134.47, 128.62, 128.26, 127.21, 127.11, 124.59, 121.59, 119.57, 116.55, 114.54, 114.49, 114.39, 114.27, 109.01, 108.95, 106.67, 106.44, 67.13 *ppm*. HR-MS (ESI) *m*/*z* calcd for C_19_H_11_Cl_2_FN_2_O_3_ (M + H)^+^ 405.0187, found 405.0199.

Synthesis of *tert*-butyl (5,7-dichloro-2-(iodomethyl)quinolin-8-yl) carbonate (**8**):

To a stirred solution of *tert*-butyl-(5,7-dichloro-2-(hydroxymethyl)quinolin-8-yl) carbonate **4b** (1 mmol) was added triphenyl phosphine (1.2 mmol), imidazole (1.3 mmol) in dichloromethane, and iodine (1.2 mmol) at room temperature. The resulting reaction mixture was stirred for 1 h. After completion of the reaction, aqueous sodium dithionite solution was added and the resulting mixture extracted with dichloromethane, the separated organic layer was dried over Na_2_SO_4_ and concentrated under reduced pressure. The crude product was purified by column chromatography (silica gel, 10% ethyl acetate in hexanes) to afford compound **8**.

^1^H-NMR (400 MHz, CDCl_3_): δ 8.42 (d, *J* = 8.4 Hz, 1H), 7.62 (s, 1H), 7.61 (d, *J* = 8.8 Hz, 1H), 4.61 (s, 2H), 1.60 (s, 9H) *ppm*. ^13^C-NMR (101 MHz, CDCl_3_): δ 160.22, 150.41, 142.71, 141.54, 134.27, 128.76, 127.40, 127.34, 124.70, 122.56, 84.43, 27.67, 5.41 *ppm*.

Synthesis of 2-(azidomethyl)-5,7-dichloroquinolin-8-yl *tert*-butyl carbonate (**9**):

To a stirred solution of *tert*-butyl-(5,7-dichloro-2-(iodomethyl)quinolin-8-yl) carbonate **8** (1 mmol) in acetone was added sodium azide (3 mmol). The resulting mixture was stirred at reflux temperature for 6 h. After completion of the reaction, the reaction mixture was cooled to room temperature then solvent was evaporated. Water was added to the crude mass and mixture extracted with dichloromethane, the separated organic layer dried over Na_2_SO_4_ and concentrated under reduced pressure. The crude product was purified by column chromatography (silica gel, 1% methanol in dichloromethane) to afford compound **9**.

^1^H-NMR (CDCl_3_, 400 MHz): δ 8.49 (d, *J* = 8.8 Hz, 1H), 8.00 (s, 1H), 7.60 (d, *J* = 8.4 Hz, 1H), 4.64 (s, 2H), 1.59 (s, 9H) *ppm*. ^13^C-NMR (CDCl_3_, 101 MHz): *δ* ^13^C-NMR (101 MHz, CDCl_3_): δ 158.07, 150.27, 144.91, 141.76, 137.04, 133.13, 126.94, 121.31, 118.93, 117.11, 84.41, 77.30, 76.98, 76.67, 55.60, 27.61 *ppm*.

Synthesis of 2-(aminomethyl)-5,7-dichloroquinolin-8-yl *tert*-butyl carbonate (**10**):

To a stirred solution of 2-(azidomethyl)-5,7-dichloroquinolin-8-yl *tert*-butyl carbonate **9** (1 mmol) in THF/H_2_O (3 mL, 9:1) was added PPh_3_ (1.2 mmol) at room temperature. The resulting reaction mixture was stirred at 60 °C for 12 h. After completion of reaction, the reaction mixture was concentrated under vacuum and 1 M aqueous HCl added to the residue. The mixture was stirred for 30 min, then the mixture was filtered to get aqueous phase. The aqueous phase was basified to pH 10 with NaHCO_3_ and extracted with dichloromethane. The combined organic layer was washed with brine, dried over anhydrous Na_2_SO_4_, and the solution was evaporated under vacuum to afford target compound **10**.

^1^H-NMR (400 MHz, CDCl_3_): δ 8.41 (d, *J* = 8.7 Hz, 1H), 7.63 (s, 1H), 7.39 (d, *J* = 8.7 Hz, 1H), 4.89 (s, 2H), 1.58 (s, 9H) *ppm*. ^13^C-NMR (101 MHz, CDCl_3_): δ 161.16, 150.50, 142.37, 140.93, 134.02, 129.02, 127.64, 127.17, 125.30, 119.75, 84.65, 77.29, 77.17, 76.97, 76.65, 64.14, 27.58 *ppm*.

Synthesis of *N*-((8-hydroxyquinolin-2-yl)methyl)-1*H*-indole-2-carboxamide hybrid analogs **12a** and **12b:**

To a stirred solution of indole-2-carboxylic acid **5a** or **5b** (0.15 mmol) in dry dichloromethane (5 mL) under an argon atmosphere at 0 °C was added EDCI (0.181 mmol) and the mixture allowed to stir for 15 min. A catalytic amount of DMAP (0.045 mmol) was then added followed by addition of 2-(aminomethyl)-5,7-dichloroquinolin-8-yl *tert*-butyl carbonate **(10)** (0.151 mmol) in dry dichloromethane (3 mL) at 0 °C, and the resulting mixture was stirred for 8 h at room temperature. After completion of the reaction (monitored by TLC), the reaction mixture was washed with water (10 mL), and the dichloromethane layer separated, dried over anhydrous sodium sulfate, filtered, concentrated, and purified by silica gel column chromatography, using 2–5% methanolic dichloromethane as eluent, to afford the corresponding Boc-protected 8-hydroxyquinoline-indole derivatives **11a** or **11b** as a solid. Products were obtained in 56–74% yield.

The above Boc-protected 8-hydroxyquinoline-indole derivatives **11a** and **11b** were each dissolved in a mixture of DCM (10 mL) and trifluoroacetic acid (TFA, 1.0 mL) and the resulting mixture stirred for 6 h until the Boc-deprotection was completed. A saturated NaHCO_3_ solution (20 mL) was then added and the resulting mixture extracted with dichloromethane. The organic layer was separated and washed with water, followed by brine solution, dried over anhydrous Na_2_SO_4_, and concentrated to afford the appropriate hybrid 8-hydroxyquinoline indole derivatives **12a** and **12b**.

*N-((5,7-Dichloro-8-hydroxyquinolin-2-yl)methyl)-1H-indole-2-carboxamide (***12a***): *^1^H-NMR (400 MHz, DMSO-*d*_6_): δ 12.01 (s, 1H), 10.71 (s, 1H), 10.07 (s, 1H), 8.52(d, *J* = 8.4 Hz, 1H), 8.07 (s, 1H), 7.93 (d, *J* = 8.8 Hz, 1H), 7.70 (d, *J* = 8.0 Hz, 1H), 7.49 (d, *J* = 8.0 Hz, 1H), 7.33 (s, 1H), 7.28 (t, *J* = 7.6 Hz, 1H), 7.12 (t, *J* = 7.6 Hz, 1H), 5.71 (s, 2H) *ppm*; ^13^C-NMR (100 MHz, DMSO-d_6_): δ 161.34, 156.89, 151.03, 138.59, 138.05, 137.00, 133.70, 127.14, 126.97, 126.25, 125.41, 122.61, 122.08, 120.77, 113.06, 109.39, 109.14, 106.03, 66.91 *ppm*. HR-MS (ESI) *m*/*z* calcd for C_19_H_13_Cl_2_N_3_O_2_ (M + H)^+^ 386.0379, found 386.0382.

*N-((5,7-Dichloro-8-hydroxyquinolin-2-yl)methyl)-5-methoxy-1H-indole-2-carboxamide (***12b***): *^1^H-NMR (400 MHz, DMSO-*d*_6_): δ 11.87 (s, 1H), 10.64 (s, 1H), 10.02 (s, 1H), 8.56 (d, *J* = 8.8 Hz, 1H), 7.87 (d, *J* = 8.8 Hz, 1H), 7.8 (s, 1H), 7.35 (d, *J* = 9.2 Hz, 1H), 7.2–7.19 (m, 1H), 7.11 (d, *J* = 2.4 Hz, 1H), 6.94 (dd, *J* = 8.8, 2.4 Hz, 1H), 5.67 (s, 2H), 3.74 (s, 3H) *ppm*; ^13^C-NMR (100 MHz, DMSO-*d*_6_): δ 161.26, 157.04, 154.46, 149.21, 138.68, 134.49, 133.43, 128.26, 127.47, 127.10, 124.60, 121.59, 119.58, 117.13, 116.55, 113.98, 108.68, 102.37, 66.93, 55.65 *ppm*. HR-MS (ESI) *m*/*z* calcd for C_20_H_15_Cl_2_N_3_O_3_ (M + H)^+^ 416.0485, found 416.0492.

Synthesis of (8-((*tert*-butoxycarbonyl)oxy)quinolin-2-yl)methyl-1*H*-1,2,4-triazole-1-carboxylates **14a–14c:**

To a stirred solution of an appropriate *tert*-butyl (2-(hydroxymethyl)quinolin-8-yl) carbonate (**4a–4c)** (1 mmol) in dichloromethane was added carbonylditriazole (2 mmol) at room temperature. The reaction mixture was stirred for 30 min. After completion of the reaction, water was added and the resulting mixture was extracted with dichloromethane, the separated organic layer dried over Na_2_SO_4_ and concentrated under reduced pressure to afford triazole intermediates **14a–14c**.

(8-((*tert*-Butoxycarbonyl)oxy)quinolin-2-yl)methyl-1*H*-1,2,4-triazole-1-carboxylate (**14a**):

^1^H-NMR (400 MHz, CDCl_3_): δ 8.88 (s, 1H), 8.27 (d, *J* = 8.5 Hz, 1H), 8.11 (s, 1H), 7.74 (dd, *J* = 6.6, 3.0 Hz, 1H), 7.63 (d, *J* = 8.5 Hz, 1H), 7.58–7.50 (m, 2H), 5.77 (s, 2H), 1.59 (s, 9H) *ppm*. ^13^C-NMR (101 MHz, CDCl_3_): δ 153.83, 153.68, 151.83, 147.28, 145.79, 140.60, 137.46, 128.89, 126.90, 125.61, 121.67, 119.87, 83.70, 71.17, 27.62 *ppm*.

(8-((*tert*-Butoxycarbonyl)oxy)-5,7-dichloroquinolin-2-yl)methyl-1*H*-1,2,4-triazole-1-carboxylate (**14b**):

^1^H-NMR (400 MHz, CDCl_3_): δ 8.89 (s, 1H), 8.59 (d, *J* = 8.8 Hz, 1H), 8.11 (s, 1H), 7.71 (d, *J* = 8.4 Hz, 2H), 5.77 (s, 2H), 1.57 (s, 9H) *ppm*. ^13^C-NMR (101 MHz, CDCl_3_): δ 155.61, 153.90, 150.35, 145.84, 142.78, 141.72, 134.77, 128.95, 127.86, 125.42, 120.29, 84.57, 70.38, 27.54 *ppm*.

(8-((*tert*-Butoxycarbonyl)oxy)-5,7-dibromoquinolin-2-yl)methyl-1*H*-1,2,4-triazole-1-carboxylate (**14c**):

^1^H-NMR (400 MHz, CDCl_3_): δ 8.89 (s, 1H), 8.54 (d, *J* = 8.7 Hz, 1H), 8.08 (d, *J* = 30.9 Hz, 2H), 7.71 (d, *J* = 8.8 Hz, 1H), 5.78 (s, 2H), 1.58 (s, 9H) *ppm*. ^13^C-NMR (1010 MHz, CDCl_3_): δ 155.53, 153.88, 150.16, 147.26, 145.86, 144.92, 141.67, 137.30, 133.51, 127.12, 120.74, 118.92, 117.35, 84.52, 70.23, 27.55 *ppm*.

Synthesis of hybrid (8-((*tert*-butoxycarbonyl)oxy)quinolin-2-yl)methylpiperazine-1- carboxylates **16a–16c**:

An appropriate triazole intermediate (**14a–14c**) (1 mmol) was dissolved in dichloromethane (2 mL) and treated with piperazine (**15**) (1.0 mmol) at room temperature for 1 h. Completion of the reaction was monitored by TLC. Water (2 mL) was then added and the resulting mixture was extracted with dichloromethane (2 × 3 mL), the organic layer separated, dried over anhydrous Na_2_SO_4_ and concentrated to afford the appropriate Boc-protected product **(16a–16c)**. The crude product was purified by column chromatography (silica gel, 2% methanol in dichloromethane) to afford the desired product **(16a–16c**).

*(8-((tert-Butoxycarbonyl)oxy)quinolin-2-yl)methyl-piperazine-1-carboxylate (***16a***)*: ^1^H-NMR (400 MHz, DMSO-*d*_6_): δ 8.33 (d, *J* = 8.4 Hz, 1H), 7.52 (d, *J* = 8.4Hz, 1H), 7.45–7.35 (m, 2H), 7.10 (dd, *J* = 7.2, 1.2 Hz, 1H), 5.33 (s, 2H), 3.37 (m, 4H), 2.72–2.61 (m, 4H), 1.58 (s, 9H) *ppm*; ^13^C-NMR (101 MHz, DMSO-*d*_6_): δ 155.59, 154.75, 153.35, 153.22, 137.99, 137.42, 128.43, 127.80, 119.91, 118.08, 112.07, 67.84, 45.81, 27.52 *ppm*.

*(8-((tert-Butoxycarbonyl)oxy)-5,7-dichloro-8-hydroxyquinolin-2-yl)methyl-piperazine-1-carboxylate (***16b***): *^1^H-NMR (400 MHz, CDCl_3_): δ 8.39 (d, *J* = 8.4 Hz, 1H), 7.52 (d, *J* = 8.8 Hz, 1H), 7.48 (s, 1H), 5.37 (s, 2H), 3.59 (m, 4H), 2.88 (br-s, 4H), 1.57 (s, 9H) *ppm*; ^13^C-NMR (101 MHz, CDCl_3_): δ 156.66, 153.64, 154.82, 147.78, 137.93, 134.32, 128.07, 124.18, 120.40, 120.31, 115.78, 67.22, 53.42, 45.49, 27.52 *ppm*.

*(8-((tert-Butoxycarbonyl)oxy)-5,7-dibromo-8-hydroxyquinolin-2-yl)methyl-piperazine-1-carboxylate (***16c**): ^1^H-NMR (400 MHz, DMSO-*d*_6_): δ 8.40 (d, *J* = 8.8 Hz, 1H), 7.97 (s, 1H), 7.68 (d, *J* = 8.8 Hz, 1H), 5.38 (s, 2H), 3.46 (m, 4H), 2.80 (br-s, 4H), 1.58 (s, 9H) *ppm*; ^13^C-NMR (101 MHz, DMSO-*d*_6_): δ 156.92, 154.60, 153.55 153.13, 139.44, 136.71, 133.63, 126.37, 121.45, 106.98, 106.32, 67.49, 45.01, 44.33, 27.51 *ppm*.

Synthesis of (8-hydroxyquinolin-2-yl)methyl-4-(1*H*-indole-2-carbonyl)piperazine-1-carboxylate hybrid derivatives (**18a–18h**):

To the stirred solution of an appropriate indole-2-carboxylic acid (**5a–5c**) (0.15 mmol) in dry dichloromethane (5 mL) under an argon atmosphere at 0 °C was added EDCI (0.181 mmol), and the mixture allowed to stir for 15 min. A catalytic amount of DMAP (0.045 mmol) was then added, followed by addition of an appropriate (8-((*tert*-butoxycarbonyloxy)-quinolin-2-yl)methyl piperazine-1-carboxylate (**16a–16c**) (0.151 mmol) in dry dichloromethane (3 mL) at 0 °C. The resulting mixture was stirred for 8 h at room temperature. After completion of the reaction (monitoring by TLC), the reaction mixture was washed with water (10 mL) and the dichloromethane layer separated, dried over anhydrous sodium sulfate, filtered, and concentrated. The crude material was purified by silica gel column chromatography, using 2–5% methanolic dichloromethane as eluent, to afford the corresponding (8-((*tert*-butoxycarbonyl)oxy) quinolin-2-yl)methyl-4-(1*H*-indole-2-carbonyl)piperazine-1-carboxylate hybrid derivatives **(17a–17h)**, which was dissolved in a mixture of DCM (10 mL) and TFA (1.0 mL) and stirred for 6 h until Boc-deprotection was completed. Saturated NaHCO_3_ solution (20 mL) was then added to the resulting mixture which was extracted with dichloromethane. The organic layer was separated and washed with water, followed by brine solution, dried over anhydrous Na_2_SO_4_ and concentrated to afford the corresponding (8-hydroxyquinolin-2-yl)methyl-4-(1*H*-indole-2-carbonyl)piperazine-1- carboxylate hybrid derivative **(18a–18h)**.

*(8-Hydroxyquinolin-2-yl)methyl-4-(1H-indole-2-carbonyl)piperazine-1-carboxylate (***18a***): *^1^H-NMR (400 MHz, CDCl_3_): δ 9.67 (s, 1H), 8.19 (d, *J* = 8.4 Hz, 1H), 7.65 (d, *J* = 8.0 Hz, 1H), 7.52–7.42 (m, 3H), 7.35–7.2 (m, 2H), 7.20–7.12 (m, 2H), 6.78 (s, 1H), 5.45 (s, 2H), 3.96 (s, 4H), 3.70 (s, 4H) *ppm*; ^13^C-NMR (100 MHz, CDCl_3_): δ 162.82, 154.94, 154.26, 151.88, 137.07, 127.85, 124.64, 121.87, 120.66, 120.06, 117.73, 111.71, 110.44, 105.49, 68.12, 43.74 *ppm*. HR-MS (ESI) *m*/*z* calcd for C_24_H_22_N_4_O_4_ (M + H)^+^ 431.1708, found 431.1712.

*(5,7-Dibromo-8-hydroxyquinolin-2-yl)methyl-4-(1H-indole-2-carbonyl)piperazine-1-carboxylate (***18b***): *^1^H-NMR (400 MHz, CDCl_3_): δ 9.50 (s, 1H), 8.44 (d, *J* = 8.8 Hz, 1H), 7.86 (s, 1H), 7.61 (dd, *J* = 15.2, 8.4 Hz, 2H), 7.41 (d, *J* = 8.0 Hz, 1H), 7.28 (t, *J* = 7.6 Hz, 1H), 7.13 (t, *J* = 7.6 Hz, 1H), 6.76 (s, 1H), 5.46 (s, 2H), 3.96 (s, 4H), 3.68 (s, 4H) *ppm*; ^13^C-NMR (100 MHz, CDCl_3_): δ 162.75, 156.36, 154.69, 149.35, 137.77, 137.09, 135.75, 133.78, 128.62, 127.32, 125.92, 124.69, 121.91, 121.05, 120.73, 111.78, 110.07, 105.50, 104.33, 67.38, 44.03 *ppm*. HR-MS (ESI) *m*/*z* calcd for C_24_H_20_Br_2_N_4_O_4_ (M + H)^+^ 588.9879, found 588.9885.

*(5,7-Dichloro-8-hydroxyquinolin-2-yl)methyl-4-(1H-indole-2-carbonyl)piperazine-1-carboxylate (***18c***): *^1^H-NMR (400 MHz, CDCl_3_): δ 10.08 (s, 1H), 8.41 (d, *J* = 8.8 Hz, 1H), 7.63 (d, *J* = 7.6 Hz, 1H), 7.56 (d, *J* = 8.4 Hz, 1H), 7.5 (s, 1H), 7.42 (d, *J* = 7.6 Hz, 1H), 7.27 (d, *J* = 7.6 Hz, 1H), 7.13 (t, *J* = 7.2 Hz, 1H), 6.76 (s, 1H), 5.45 (s, 2H), 3.99 (s, 4H), 3.7 (s, 4H) *ppm*. ^13^C-NMR (100 MHz, CDCl_3_): δ 162.98, 156.40, 154.75, 147.29, 137.69, 135.95, 134.42, 128.61, 128.14, 127.22, 124.54, 124.13, 121.84, 120.68, 120.61, 120.45, 115.68, 111.89, 105.52, 67.48, 43.82 *ppm*. HR-MS (ESI) *m*/*z* calcd for C_24_H_20_Cl_2_N_4_O_4_ (M + H)^+^ 499.0925, found 499.0929.

*(8-Hydroxyquinolin-2-yl)methyl-4-(5-methoxy-1H-indole-2-carbonyl)piperazine-1-carboxylate (***18d***): *^1^H-NMR (400 MHz, DMSO-*d*_6_): δ 11.46 (s, 1H), 9.64 (s, 1H), 8.34 (d, *J* = 8.4 Hz, 1H), 7.58 (d, *J* = 8.4 Hz, 1H), 7.45–7.38 (m, 2H), 7.33 (d, *J* = 8.8 Hz, 1H), 7.12 (d, *J* = 6.8 Hz, 1H), 7.07 (s, 1H), 6.86 (dd, *J* = 8.8, 2.4 Hz, 1H), 6.75 (s, 1H), 5.38 (s, 2H), 3.81 (br-s, 4H), 3.75 (s, 3H), 3.63 (br-s, 2H), 3.56 (br-s, 2H) *ppm*; ^13^C-NMR (100 MHz, DMSO-*d*_6_): δ 162.65, 155.37, 154.78, 154.18, 153.37, 138.03, 137.43, 131.66, 130.37, 128.50, 127.83, 127.52, 120.01, 118.13, 114.89, 113.36, 112.13, 104.51, 102.31, 68.09, 55.66, 44.04 *ppm*. HR-MS (ESI) *m*/*z* calcd for C_25_H_24_N_4_O_5_ (M + H)^+^ 461.1811, found 461.1813.

*(5,7-Dibromo-8-hydroxyquinolin-2-yl)methyl-4-(5-methoxy-1H-indole-2-carbonyl)piperazine-1-carboxylate (***18e***): *^1^H-NMR (400 MHz, DMSO-*d*_6_): δ 11.46 (s, 1H), 8.48 (d, *J* = 8.8 Hz, 1H), 8.05 (s, 1H), 7.8 (d, *J* = 8.8 Hz, 1H), 7.33 (d, *J* = 9.2 Hz, 1H), 7.07 (d, *J* = 2.0 Hz, 1H), 6.85 (dd, *J* = 8.8, 2.4 Hz, 1H), 6.75 (s, 1H), 5.45 (s, 2H), 3.821 (s, 4H), 3.75 (s, 3H), 3.65 (br-s, 4H) *ppm*. ^13^C-NMR (100 MHz, DMSO-*d*_6_): δ 162.66, 157.61, 154.62, 154.18, 151.04, 138.57, 136.89, 133.57, 131.66, 130.35, 127.51, 126.16, 121.75, 114.90, 113.36, 109.33, 105.98, 104.52, 102.30, 67.54, 55.67 *ppm*. HR-MS (ESI) *m*/*z* calcd for C_25_H_22_Br_2_N_4_O_5_ (M + H)^+^ 618.9998, found 619.0000.

*(5,7-Dichloro-8-hydroxyquinolin-2-yl)methyl-4-(5-methoxy-1H-indole-2-carbonyl)piperazine-1-carboxylate (***18f***): *^1^H-NMR (400 MHz, DMSO-*d*_6_): δ 11.46 (s, 1H), 8.54 (d, *J* = 8.8 Hz, 1H), 7.80 (s, 1H), 7.78 (d, *J* = 8.8 Hz, 1H), 7.33 (d, *J* = 8.8 Hz, 1H), 7.06 (d, *J* = 2.4 Hz, 1H), 6.85 (dd, *J* = 8.8, 2.4 Hz, 1H), 6.74 (s, 1H), 5.44 (s, 2H), 4.04 (s, 2H), 3.82 (s, 2H), 3.75 (s, 3H), 3.66 (s, 2H), 3.54 (s, 2H) *ppm*; ^13^C-NMR (100 MHz, DMSO-*d*_6_): δ 162.66, 162.61, 157.66, 154.61, 154.17, 149.09, 138.58, 134.32, 131.65, 131.50, 130.32, 130.18, 128.09, 127.49, 127.45, 124.49, 121.23, 119.58, 116.43, 114.88, 113.34, 113.29, 104.49, 104.45, 102.25, 67.63, 44.09, 40.54, 40.33, 40.12, 39.91, 39.70, 39.49, 39.28 *ppm*. HR-MS (ESI) *m*/*z* calcd for C_25_H_22_Cl_2_N_4_O_5_ (M + H)^+^ 529.1026, found 529.1031.

*(5,7-Dichloro-8-hydroxyquinolin-2-yl)methyl-4-(5-fluoro-1H-indole-2-carbonyl)piperazine-1-carboxylate (***18g***): *^1^H-NMR (400 MHz, DMSO-*d*_6_): δ 11.73 (s, 1H), 8.52 (d, *J* = 8.8 Hz, 1H), 7.80 (s, 1H), 7.77 (d, *J* = 8.8 Hz, 1H), 7.43 (dd, *J* = 8.8, 4.8 Hz, 1H), 7.37 (d, *J* = 10.0 Hz, 1H), 7.05 (t, *J* = 9.2 Hz, 1H), 6.83 (s, 1H), 5.44 (s, 2H), 3.82 (s, 4H), 3.66 (br-s, 2H), 3.55 (br-s, 2H) *ppm*. ^13^C-NMR (100 MHz, DMSO-*d*_6_): δ 162.35, 158.72, 157.60, 156.40, 154.62, 149.28, 138.66, 134.30, 133.13, 131.83, 128.11, 127.33, 127.22, 124.50, 121.22, 119.42, 116.47, 113.77, 113.67, 112.57, 112.31, 106.04, 105.81, 104.72, 104.67, 67.66, 43.92 *ppm*. HR-MS (ESI) *m*/*z* calcd for C_24_H_19_Cl_2_FN_4_O_4_ (M + H)^+^ 517.0829, found 517.0833.

*(5,7-Dibromo-8-hydroxyquinolin-2-yl)methyl-4-(5-fluoro-1H-indole-2-carbonyl)piperazine-1-carboxylate (***18h***): *^1^H-NMR (400 MHz, DMSO-*d*_6_): δ 11.72 (s, 1H), 9.65 (s, 1H), 8.35 (d, *J* = 8.4 Hz, 1H), 7.58 (d, *J* = 8.4 Hz, 1H), 7.45–7.35 (m, 4H), 7.12 -7.03 (m, 2H), 6.83 (d, *J* = 1.6 Hz, 1H), 5.38 (s, 2H), 3.81 (br-s, 4H), 3.63 (br-s, 2H), 3.54 (br-s, 2H) *ppm*. ^13^C-NMR (101 MHz, DMSO-*d*_6_): δ 162.33, 158.71, 156.39, 155.35, 154.78, 153.35, 138.01, 137.44, 133.12, 131.83, 128.50, 127.84, 127.33, 127.22, 120.01, 118.14, 113.77, 113.67, 112.57, 112.31, 112.14, 106.05, 105.82, 104.74, 104.69, 68.09, 44.0. HR-MS (ESI) *m*/*z* calcd for C_24_H_21_FN_4_O_4_ (M + H)^+^ 449.1621, found 449.1616.

### 3.3. Methodology for the In Vitro Self-Induced Aβ_1–42_ Aggregation Assay

The thioflavin T (ThT)-fluorescence assay was used to measure the inhibition of Aβ aggregation [42]. Aβ_1–42_ (Adooq Bioscience, CA, USA) was pretreated with 1 mL of hexafluoroisopropanol (HFIP) to afford a stock solution, which was aliquoted into small samples. The solvent was evaporated at room temperature, and samples were stored at −80 °C. For Aβ_1–42_ aggregation inhibition experiments, phosphate buffer (pH 7.4) was added to the Aβ stock solution to afford a 50 µM concentration before use. A mixture of the Aβ_1–42_ peptide (10 µL, 25 µM final concentration) with or without the test compound (10 µL; 0.3, 1.0, 3, 9, and 27 µM) was incubated at 37 °C for 48 h. Blanks using phosphate buffer (pH 7.4) instead of Aβ, with or without test compound, were also assessed. Then 50 mM glycine-NaOH buffer (pH 8.0) containing ThT (5 µM) was added to 20 µL of the sample to afford a final volume of 200 µL. The fluorescence intensities were recorded (excitation, 450 nm; emission, 485 nm). The fluorescence intensities were background corrected to the no enzyme control and 100% of intensity reflected no inhibition of aggregation. To obtain the normalized amyloid aggregation inhibition relative to the control group, the amyloid aggregation/cell values were further divided by the total amyloid fluorescence of the control group. Using GraphPad Prism, the amyloid aggregation values (relative to control group) were entered along the corresponding concentrations for the triplicates and then a log transformation was obtained. Using the dose–response simulation module in GraphPad Prism, nonlinear regression fit (curve) was then generated for each small molecule with various concentrations used. Curves were then compared to determine if they were statistically different, by performing the best-fit values of selected unshared parameters between data sets and extra sum-of-squares F test, allowing for selection of the simpler model unless the *P* value was less than 0.05. The logEC_50_ output parameter was then selected to obtain the EC_50_ for each of the dose–response curves for the small molecules.

### 3.4. Methodology for the In Vitro Metal-Induced Aβ_1–42_ Aggregation Assay

For the inhibition of copper- and zinc-mediated Aβ_1–42_ aggregation, 20 µM HEPES (pH 6.6) in 150 µM NaCl was added to the Aβ_1–42_ stock solution to afford a 25 µM solution. Mixtures of the peptide (10 µL, 25 µM final concentration) with or without Cu^2+^ or Zn^2+^ (10 µL, 25 μM final concentration) and the test compound (10 µL, 50 μM final concentration) were incubated at 37 °C for 24 h. Then 50 mM glycine-NaOH buffer (pH 8.0) containing ThT (5 µM) was added to 20 µL of the sample and diluted to afford a final volume of 200 µL. Fluorescence intensities of the solutions were recorded (excitation, 450 nm; emission, 485 nm). The percentage of aggregation was calculated by the expression (1 − IFi/IFc) × 100, in which IFi and IFc are the fluorescence intensities obtained for Aβ in the presence and absence of inhibitors, respectively.

### 3.5. Methodology for the Metal-Chelation Study

The chelating studies were performed with a UV–vis spectrophotometer. The absorption spectra of test compound (50 μM, final concentration) alone or in the presence of CuSO_4_, FeSO_4_, or ZnCl_2_ (50 μM, final concentration) in buffer (20 mM HEPES, 150 mM NaCl, pH 7.4) were incubated for 30 min and then recorded at room temperature, respectively. For the stoichiometry of the test compound–Cu^2+^ complex or the test compound–Zn^2+^ complex, a fixed amount of test compound (50 μM) was mixed with increasing amounts of copper ion or Zn ion (0**–**100 μM), and the UV–vis difference spectra were analyzed to determine the ratio of ligand/metal ion in the complex.

### 3.6. Methodology for In Vitro HEK-Tau and SY5Y-APP_Sw_ Cell Aggregation Assays

Human Embryonic Kidney cells that overexpress tau protein (HEK-tau) cells or SY5Y neuroblastoma cells that express a familial-AD mutant of amyloid precursor protein (SY5Y-APP_Sw_ cells) were each seeded in 96-well plates at 8000 cells per well. After 24 h, cells were supplemented with test compound at 1 µM after diluting with medium. Vehicle only control was also included for each experiment. The cells were cultured for an additional 48 h at 37 °C. Both the cell lines were able to reach approximately 80% confluency in 48 h. Cells were assessed for amyloid deposits by staining with 0.1% *w/v* ThT, counterstaining nuclei with DAPI, and calculating mean ThT fluorescence signal per nucleus over multiple fields.

### 3.7. Methodology for Molecular Docking

Molecular docking was performed using Schrodinger software Maestro 11.4 suite. The ligands were prepared using the LigPrep module. The protein crystal structure PDB ID: 1IYT was downloaded from the RCSB Protein Data Bank (https://www.rcsb.org/). The downloaded protein crystal structure was prepared in Protein Preparation Wizard wherein the missing hydrogens were added and bond orders assigned. A grid for docking the ligand was generated with the centroid of the co-crystallized ligand from PDB ID: 1IYT as the center of the grid with no constraints. The prepared ligands **18d** and **18f** were docked into this grid using Glide module. Molecular docking was performed at XP precision and results were analyzed in the Maestro visualizer.

## Figures and Tables

**Table 1:** EC_50_ (µM) values of compound **1**, and hybrid 8-hydroxyquinoline-indole derivatives **7a–7e, 12a–12b** and **18a–18h** against self-induced Aβ_1–42_ aggregation in thioflavin T (ThT)-fluorescence assays.

Compd.	1	7a	7b	7c	7d	7e	12a	12b
Aβ_1–42_	9.95	4.26	9.28	3.22	6.34	9.52	>10	>10
**Compd.**	**18a**	**18b**	**18c**	**18d**	**18e**	**18f**	**18g**	**18h**
Aβ_1–42_	>10	>10	1.72	1.48	2.49	1.08	>10	2.58

## Supplementary Materials

The following are available online.
