# Supplementary material for: Design and Synthesis of Novel Hybrid 8-Hydroxy Quinoline-Indole Derivatives as Inhibitors of Aβ Self-Aggregation and Metal Chelation-Induced Aβ Aggregation
Source: Molecules. 2020 Aug 8;25(16):3610. doi: 10.3390/molecules25163610 (PMC7463714; doi:10.3390/molecules25163610)
Supplement: Supplementary file 1 [file molecules-25-03610-s001.pdf]

SUPPORTING INFORMATION

Design and Synthesis of Novel Hybrid 8-Hydroxy Quinoline-Indole Derivatives as Inhibitors of Aβ Self-Aggregation and Metal Chelation-Induced Aβ Aggregation

Suresh K. Bowroju [1], Nirjal Mainali [2], Srinivas Ayyadevara [3], Narsimha R. Penthala [1], Sesha Krishnamachari [3], Samuel Kakraba [2], Robert J. Shmookler Reis[2-4] and Peter A. Crooks [1,*]

1 Department of Pharmaceutical Sciences, College of Pharmacy, University of Arkansas for Medical Sciences, Little Rock, AR-72205.
2 Bioinformatics Program, University of Arkansas at Little Rock and University of Arkansas for Medical Sciences, Little Rock AR-72205.
3 Central Arkansas Veterans Healthcare Service, University of Arkansas for Medical Sciences, Little Rock AR-72205.
4 Department of Geriatrics, College of Medicine, University of Arkansas for Medical Sciences, Little Rock, AR-72205.
* Correspondence: PACrooks@uams.edu.

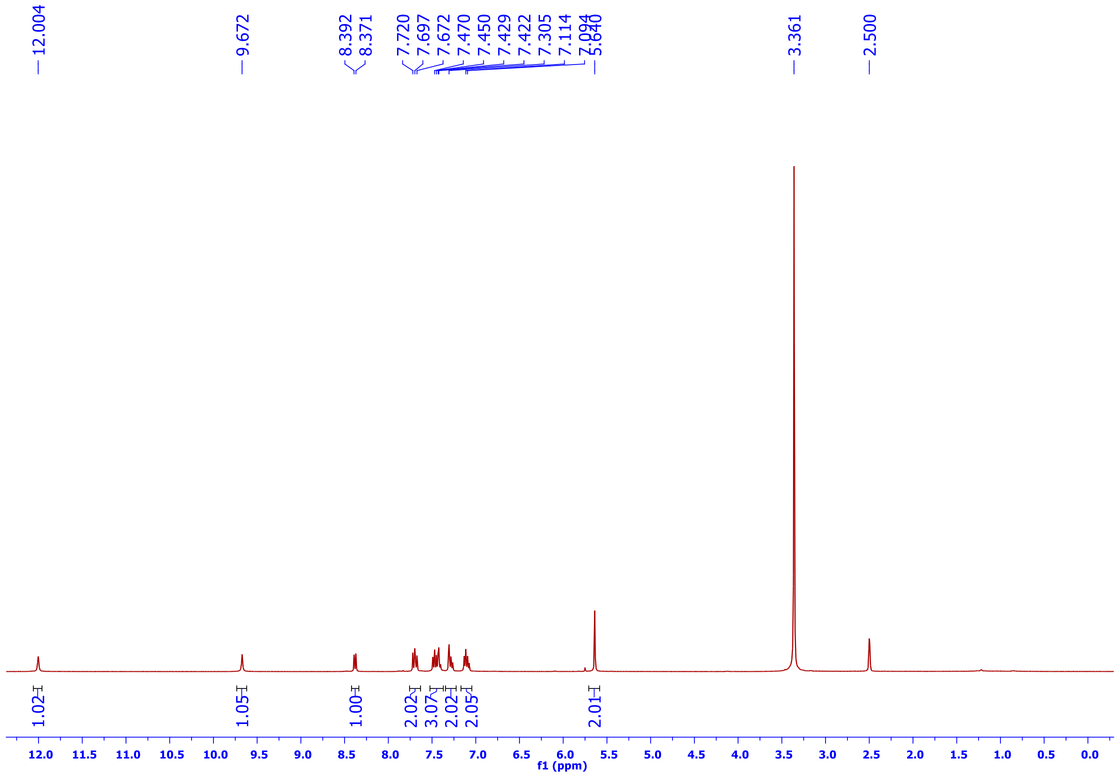

^{1}H NMR spectrum of compound **7a**

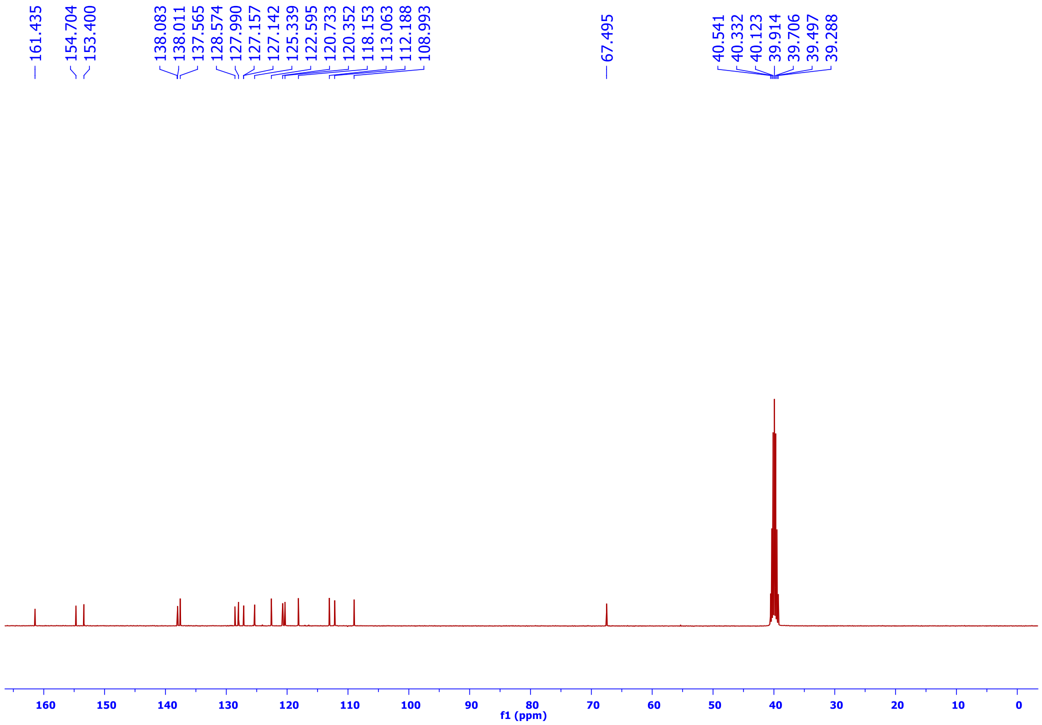

^{13}C NMR spectrum of compound **7a**

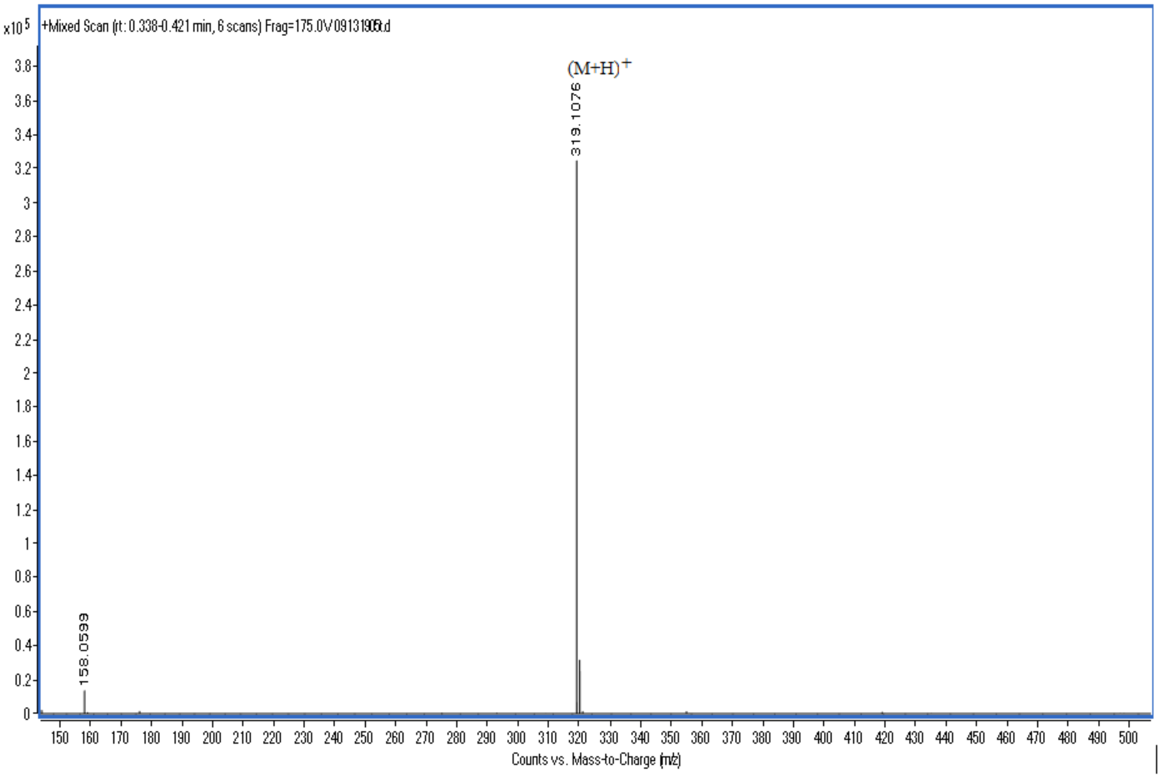

HRMS spectrum of compound **7a**

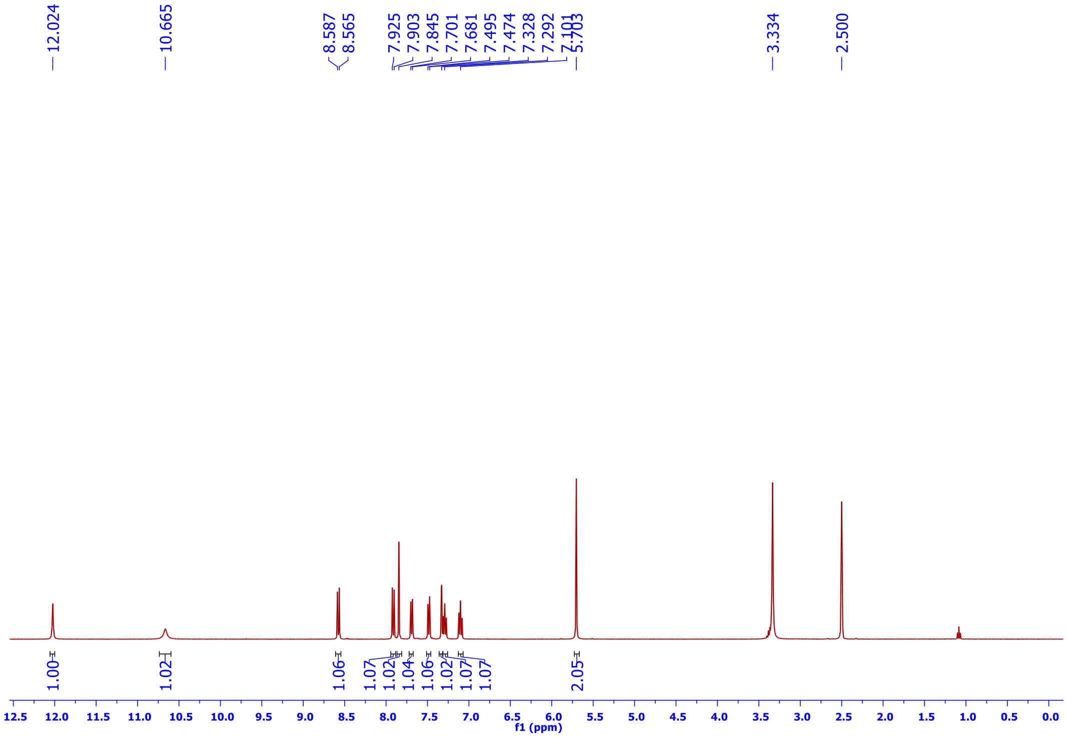

^{1}H NMR spectrum of compound **7b**

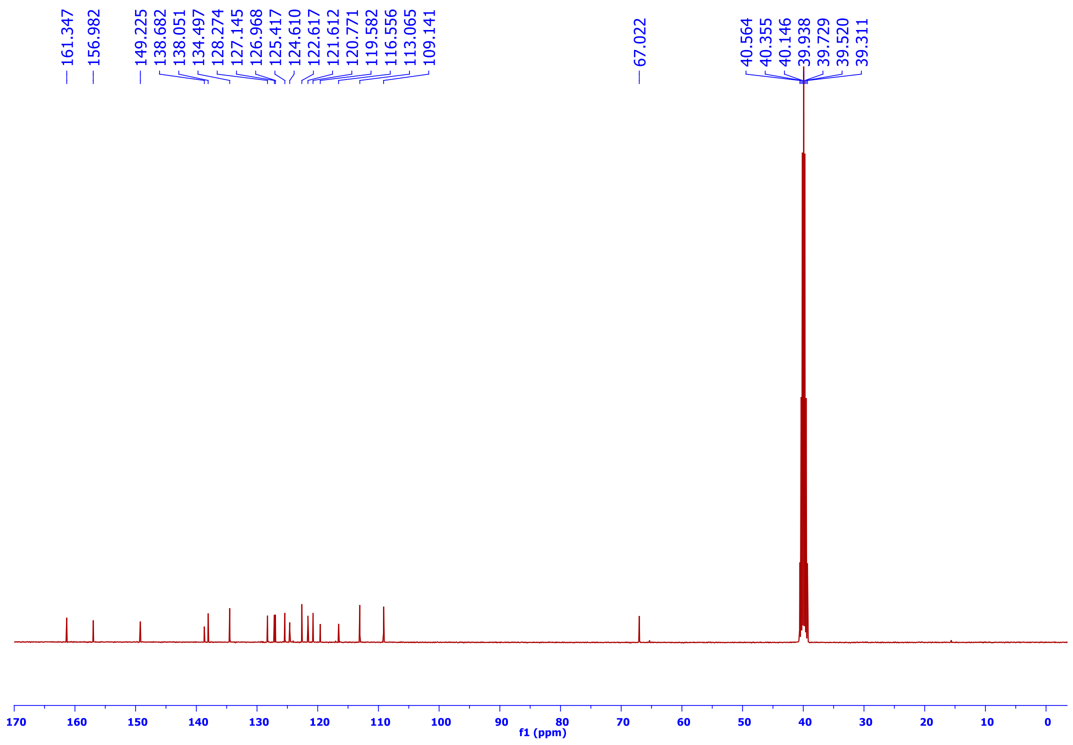

^{13}C NMR spectrum of compound **7b**

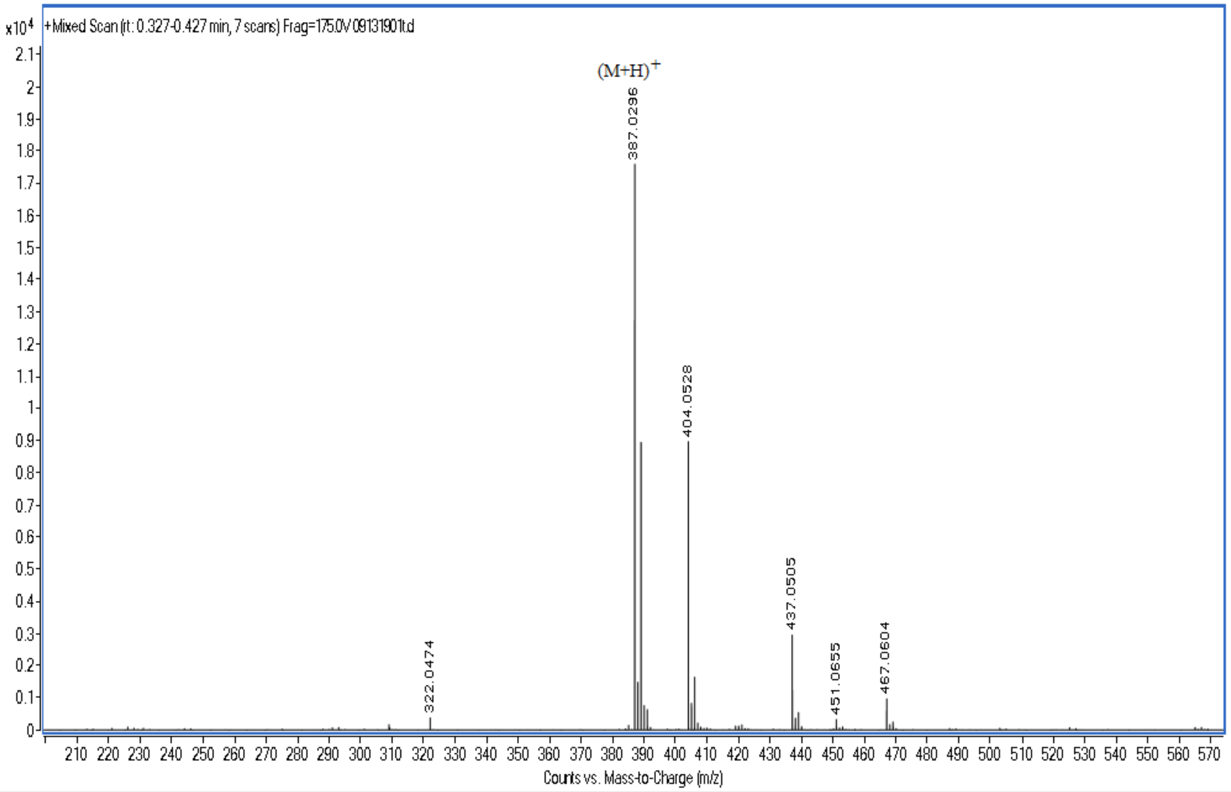

HRMS spectrum of compound **7b**

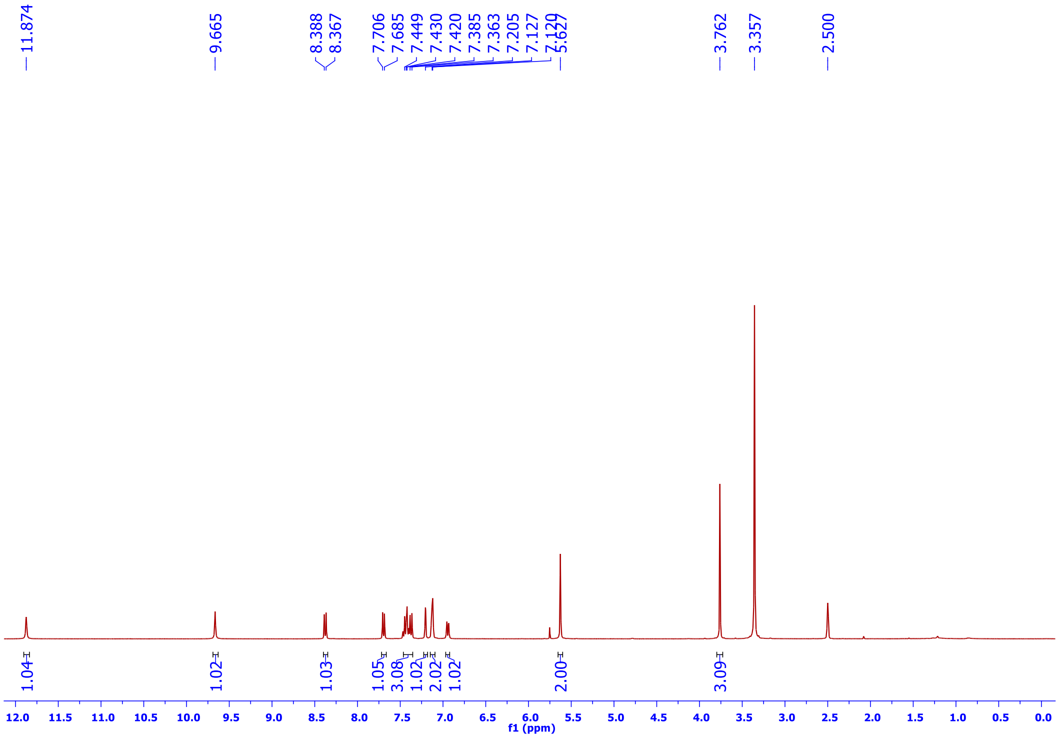

^{1}H NMR spectrum of compound **7c**

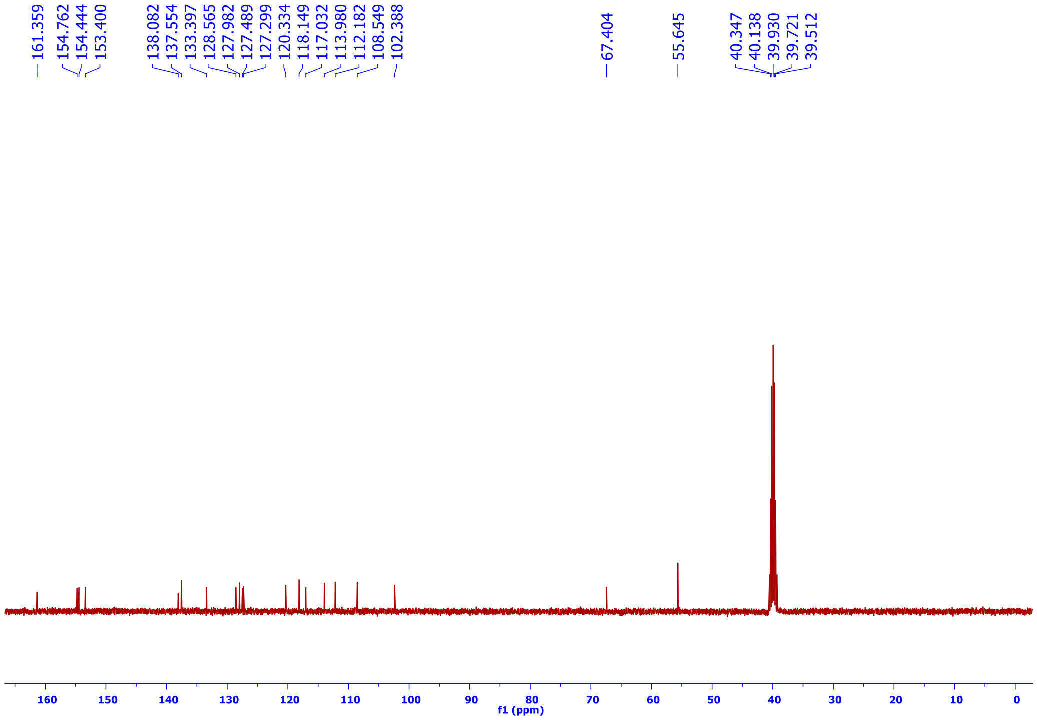

^{13}C NMR spectrum of compound **7c**

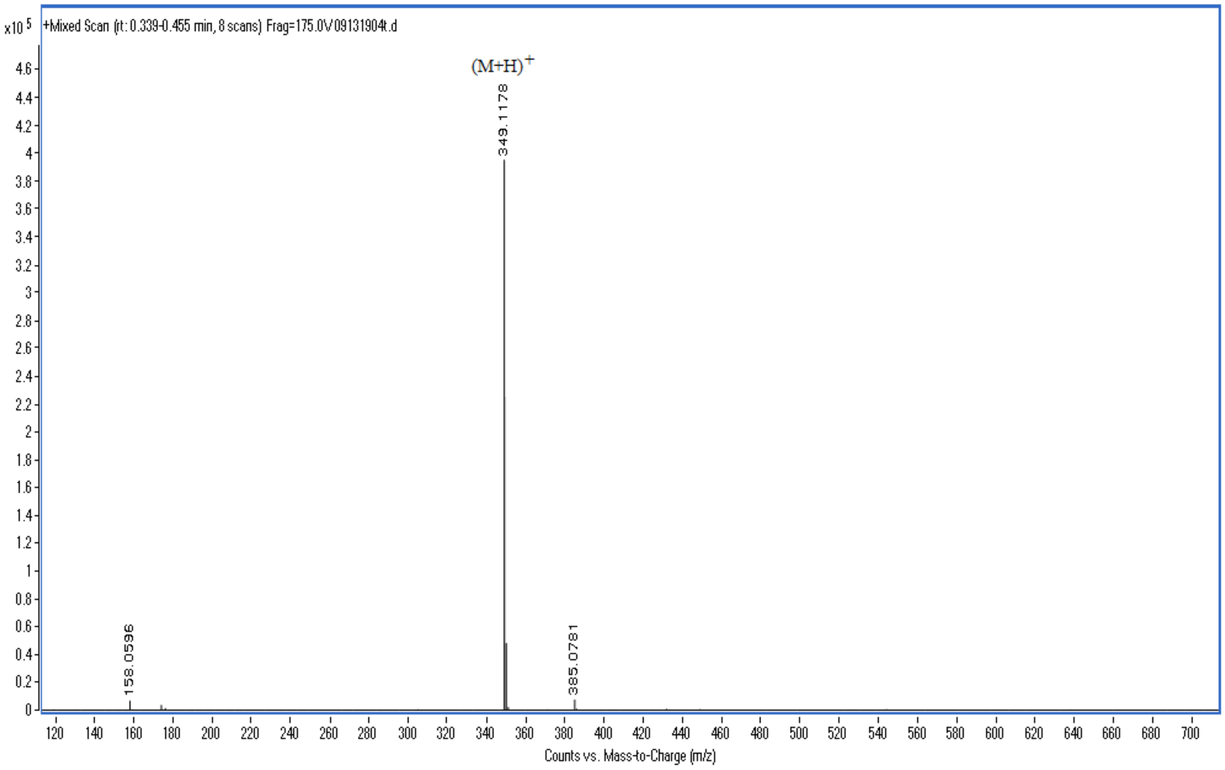

HRMS spectrum of compound **7c**

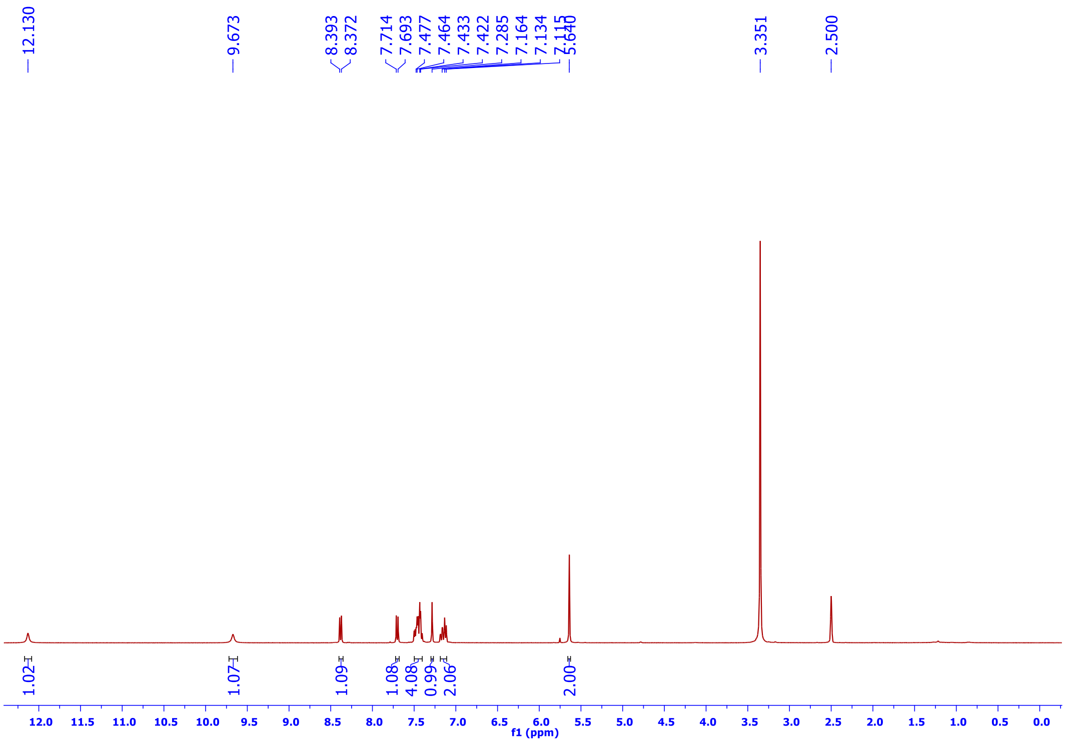

^{1}H NMR spectrum of compound **7d**

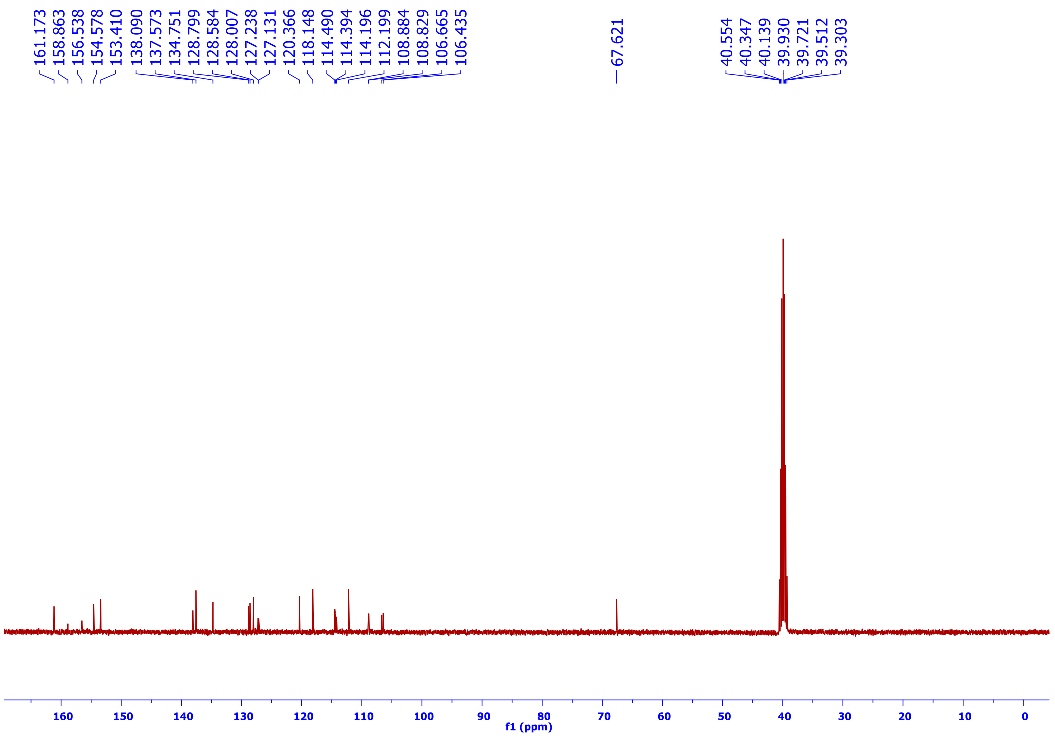

^{13}C NMR spectrum of compound **7d**

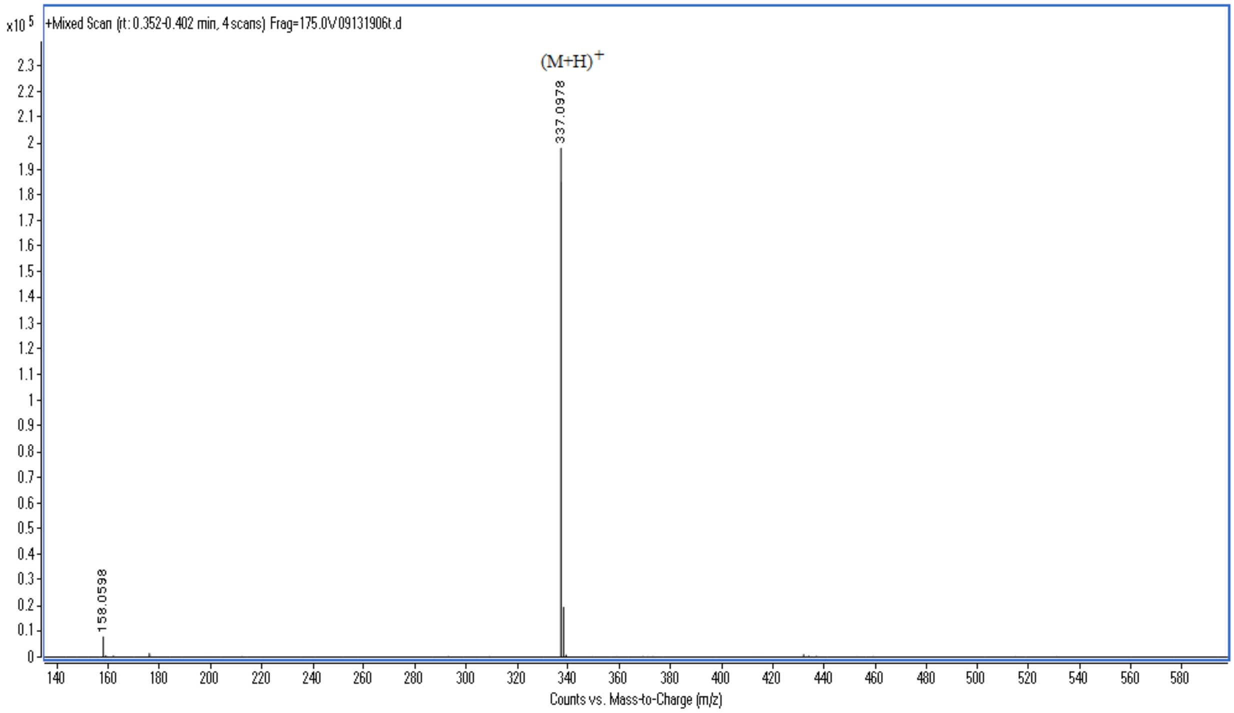

HRMS spectrum of compound **7d**

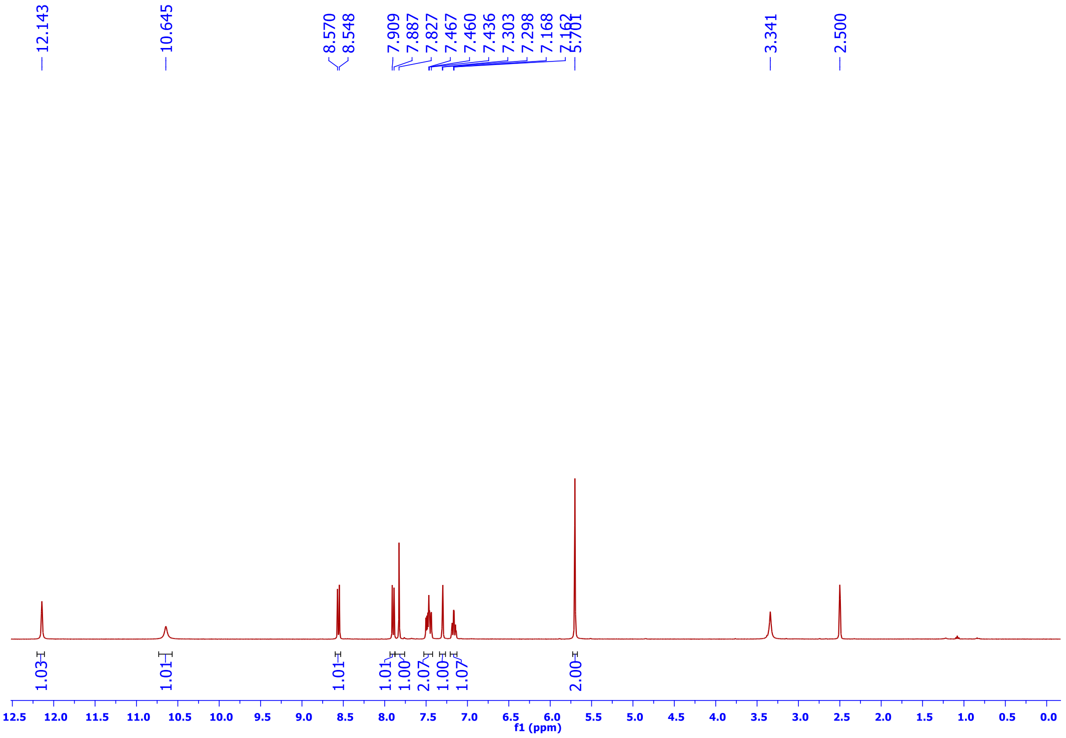

^{1}H NMR spectrum of compound **7e**

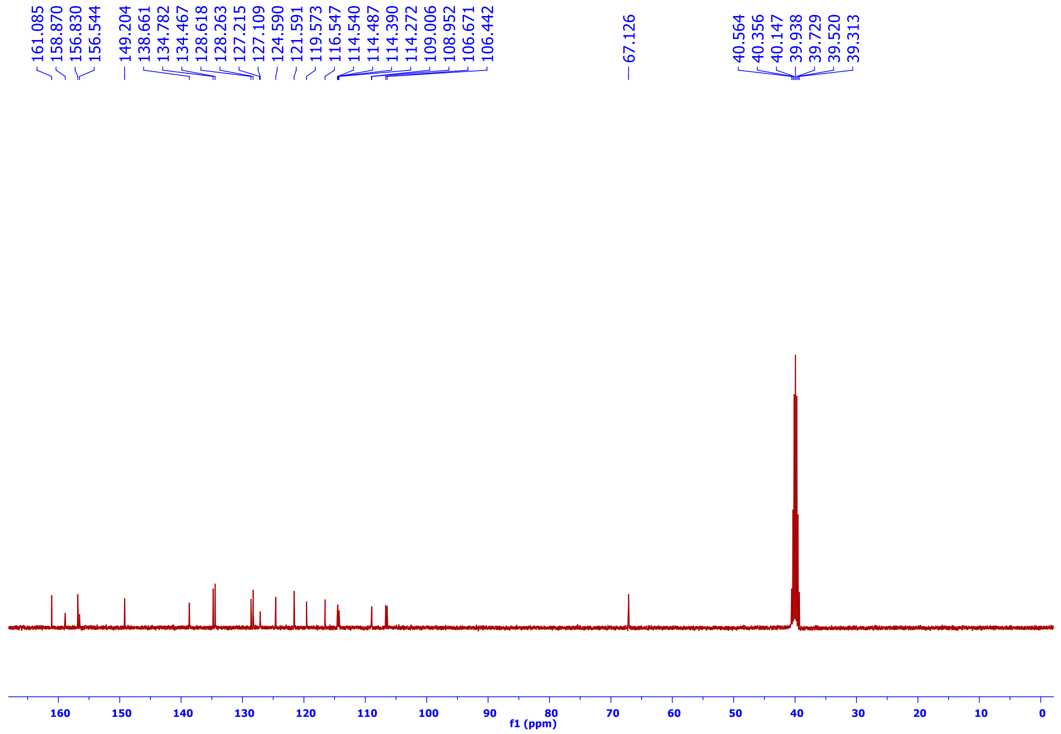

^{13}C NMR spectrum of compound **7e**

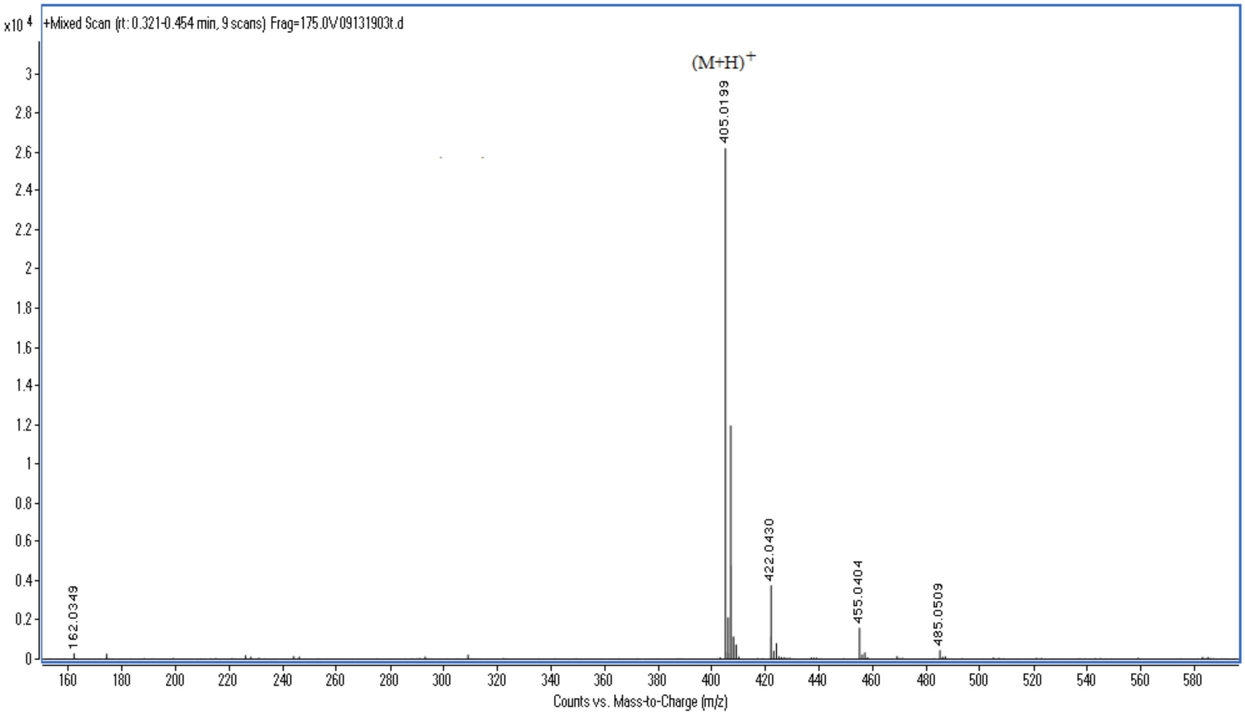

HRMS spectrum of compound **7e**

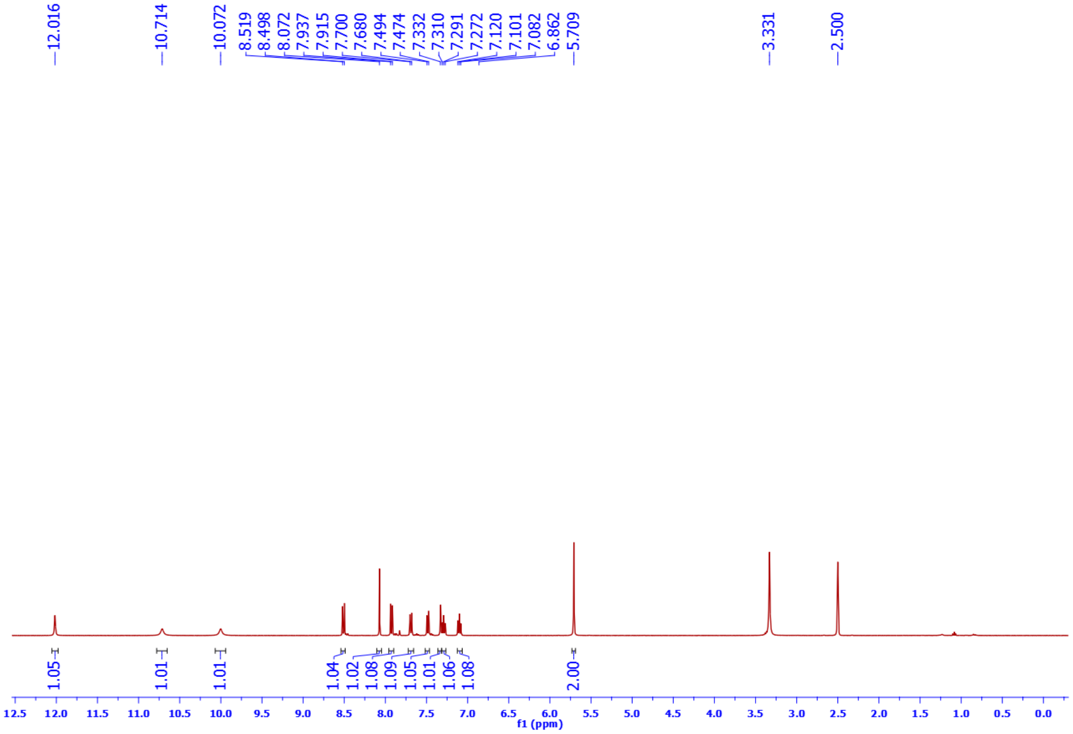

^{1}H NMR spectrum of compound **12a**

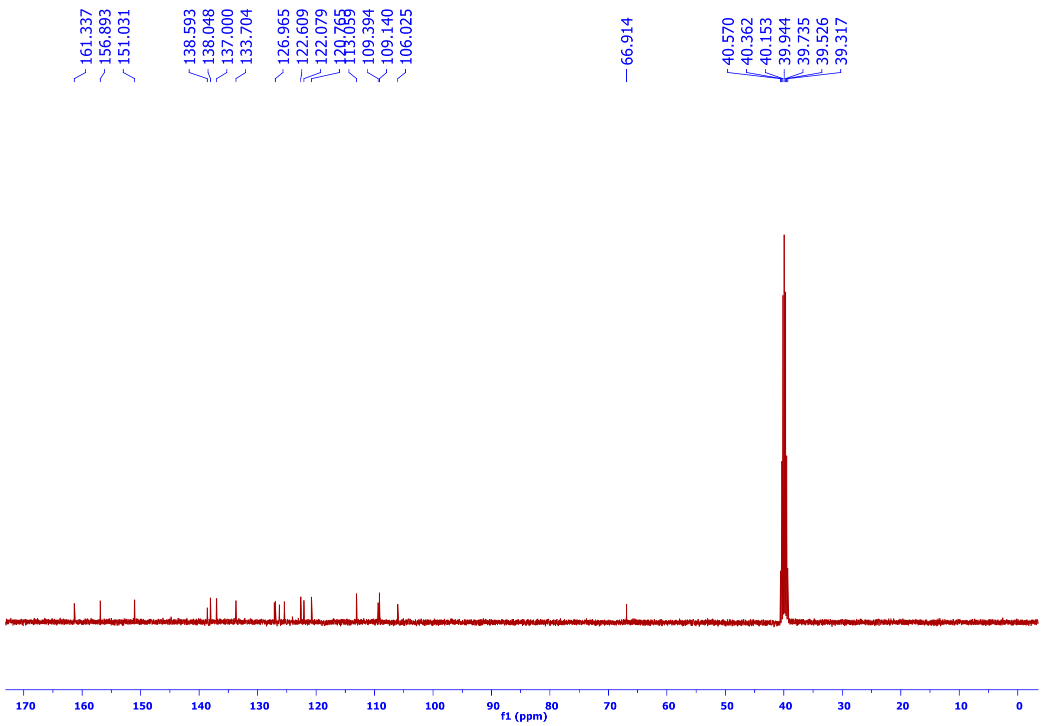

^{13}C NMR spectrum of compound **12a**

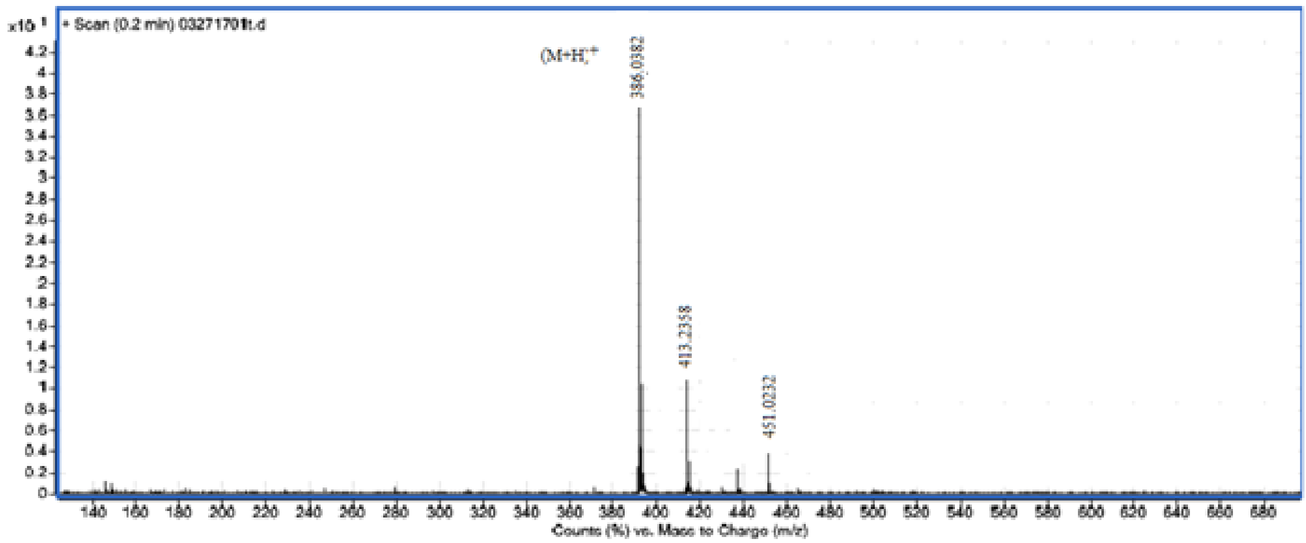

HRMS spectrum of compound **12a**

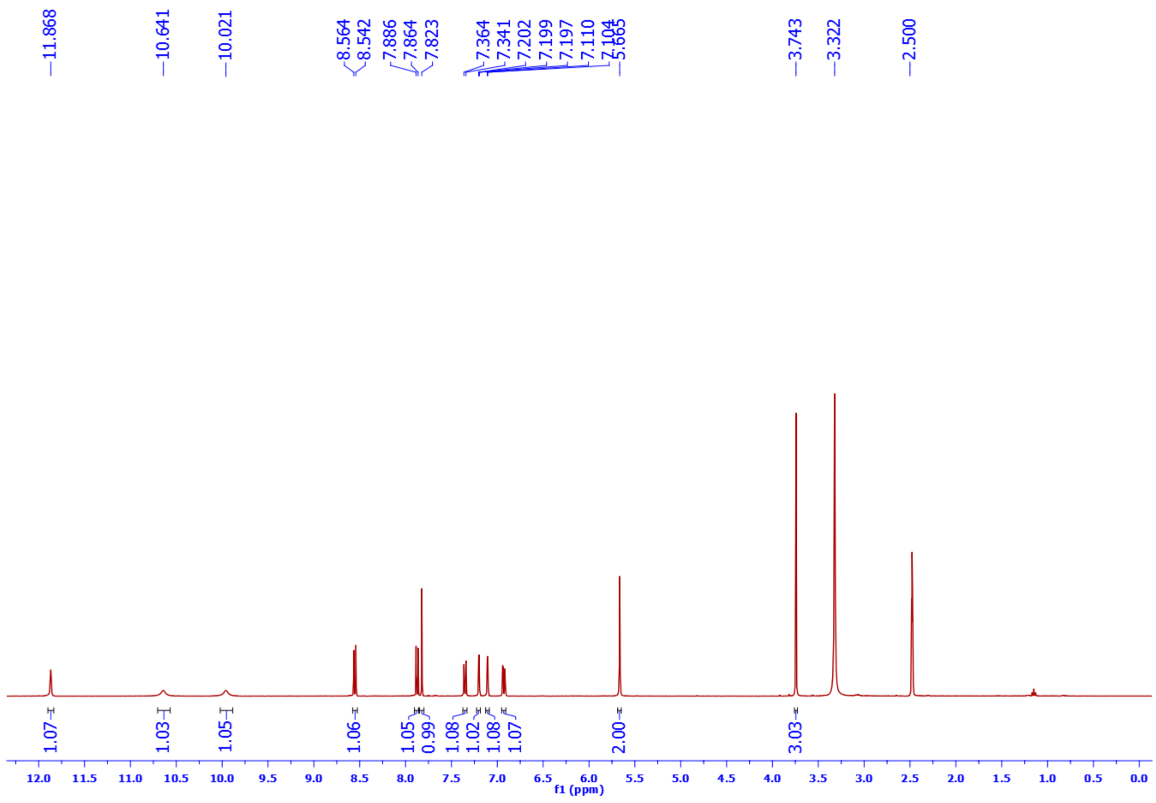

^{1}H NMR spectrum of compound **12b**

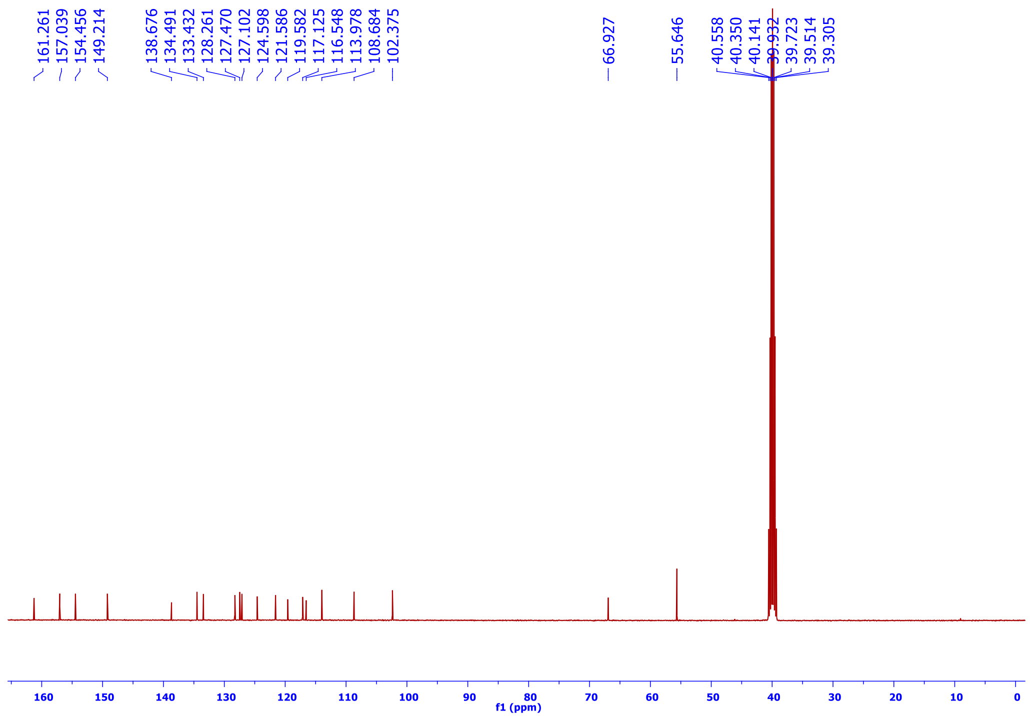

^{13}C NMR spectrum of compound **12b**

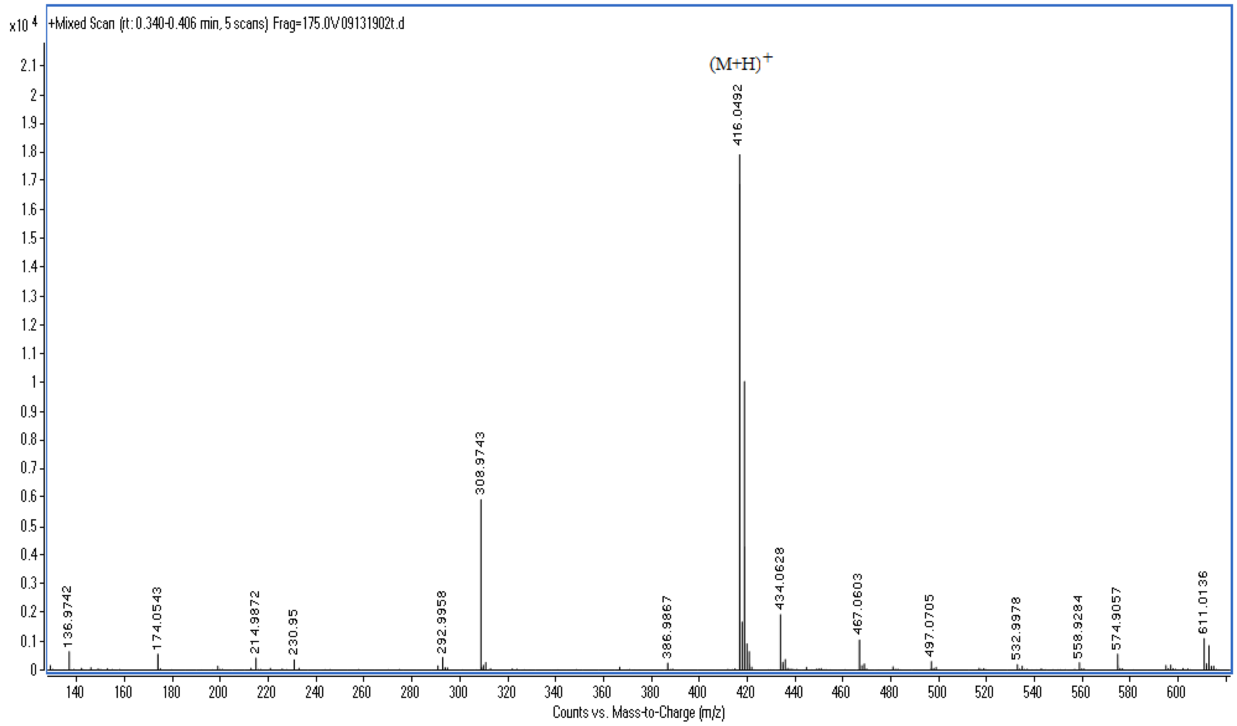

HRMS spectrum of compound **12b**

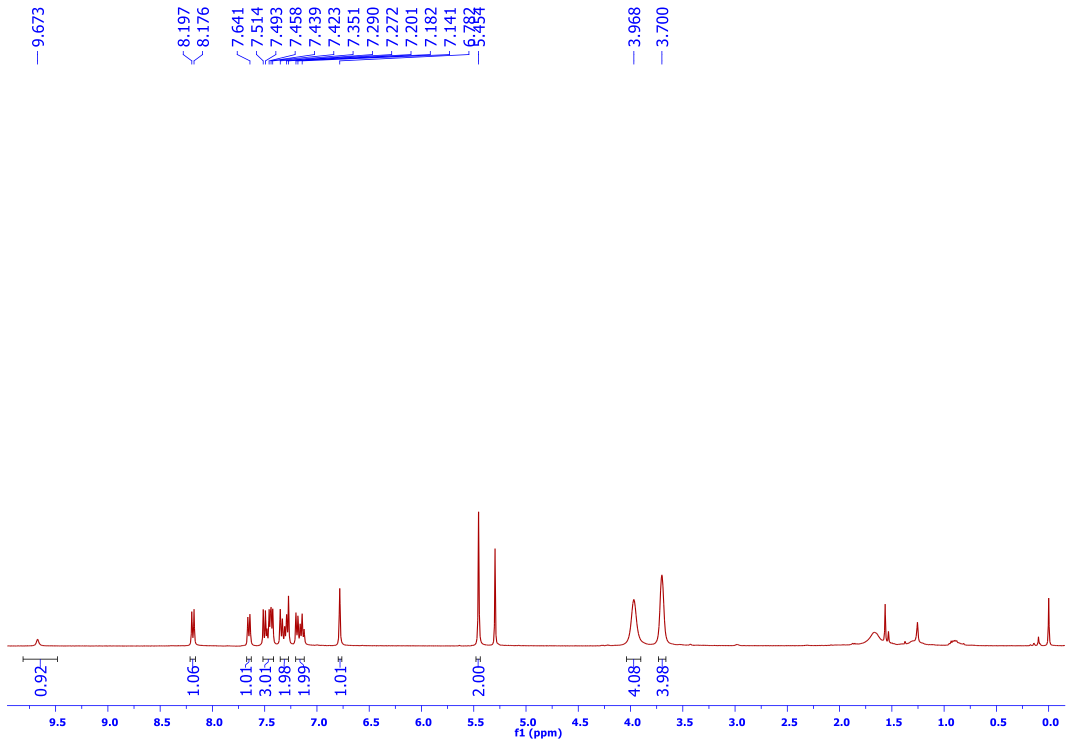

^{1}H NMR spectrum of compound **18a**

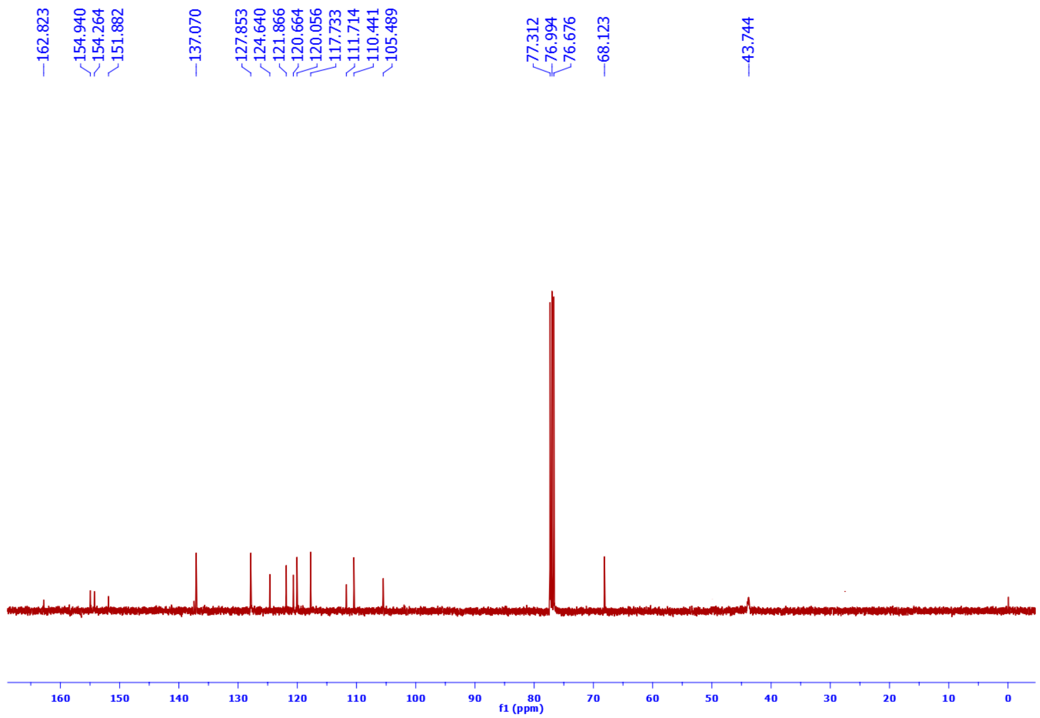

^{13}C NMR spectrum of compound **18a**

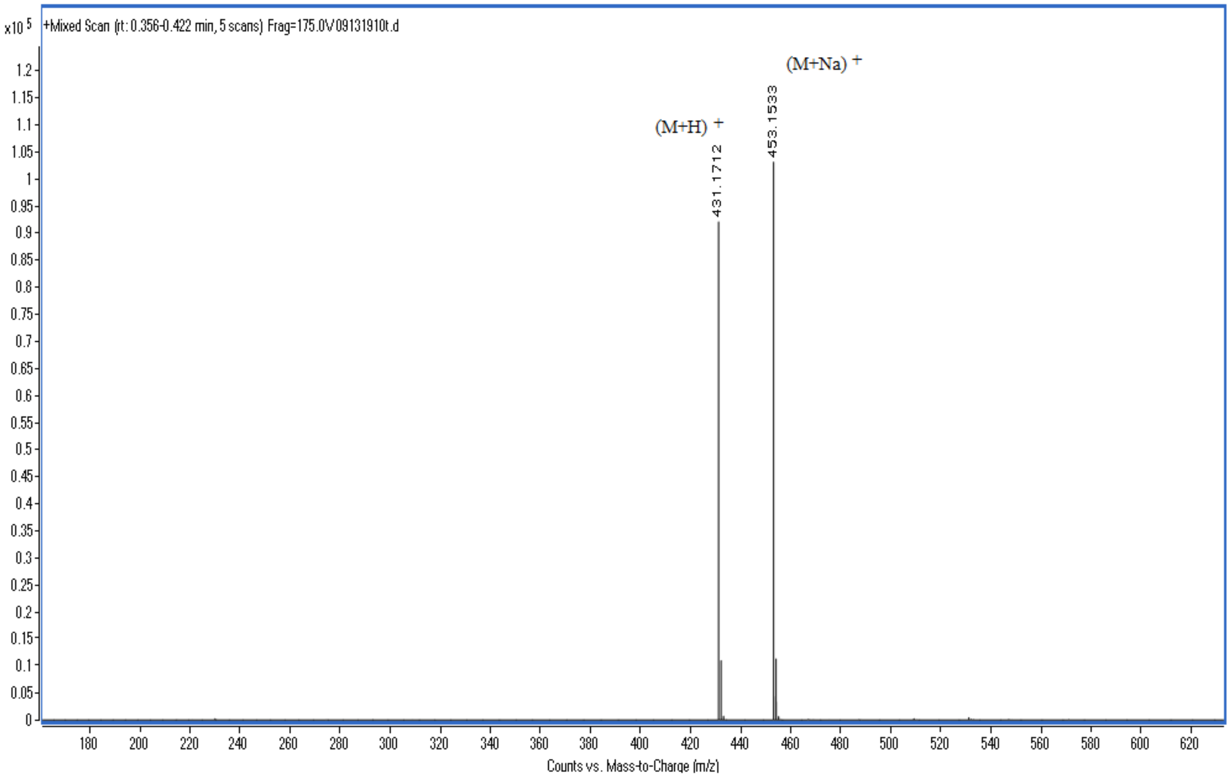

HRMS spectrum of compound **18a**

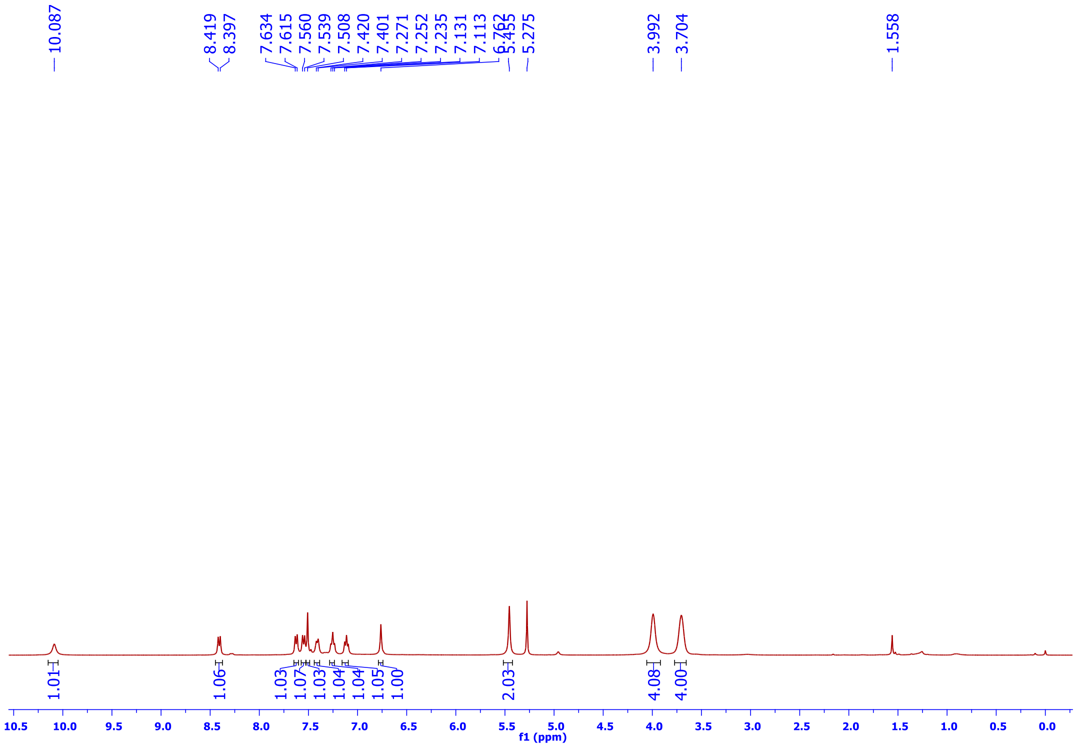

^{1}H NMR spectrum of compound **18b**

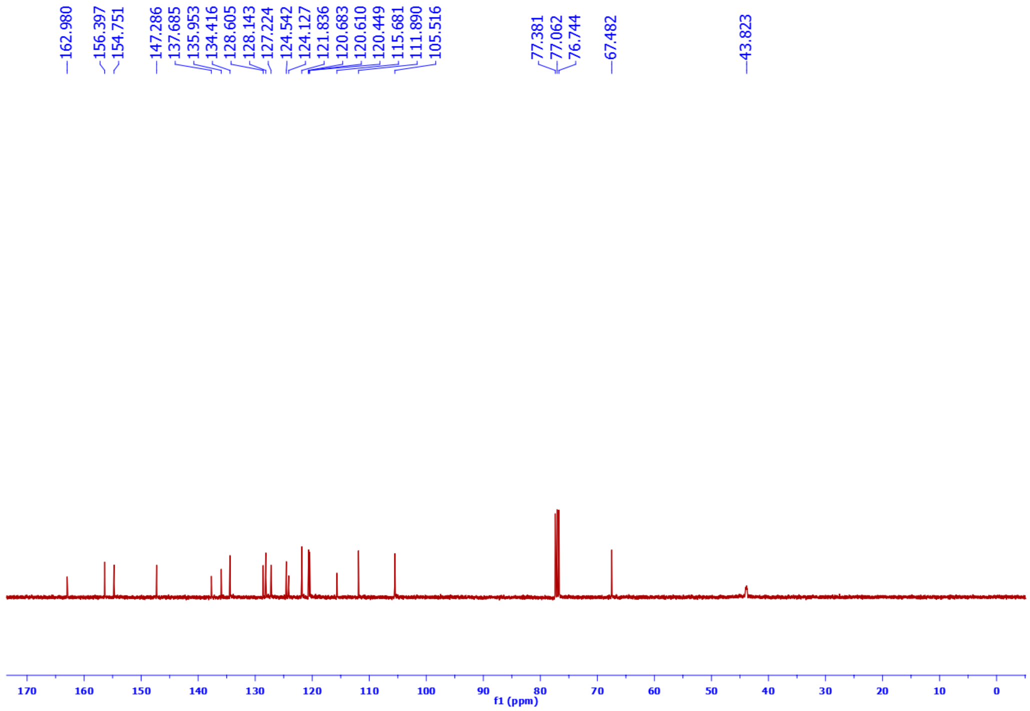

^{13}C NMR spectrum of compound **18b**

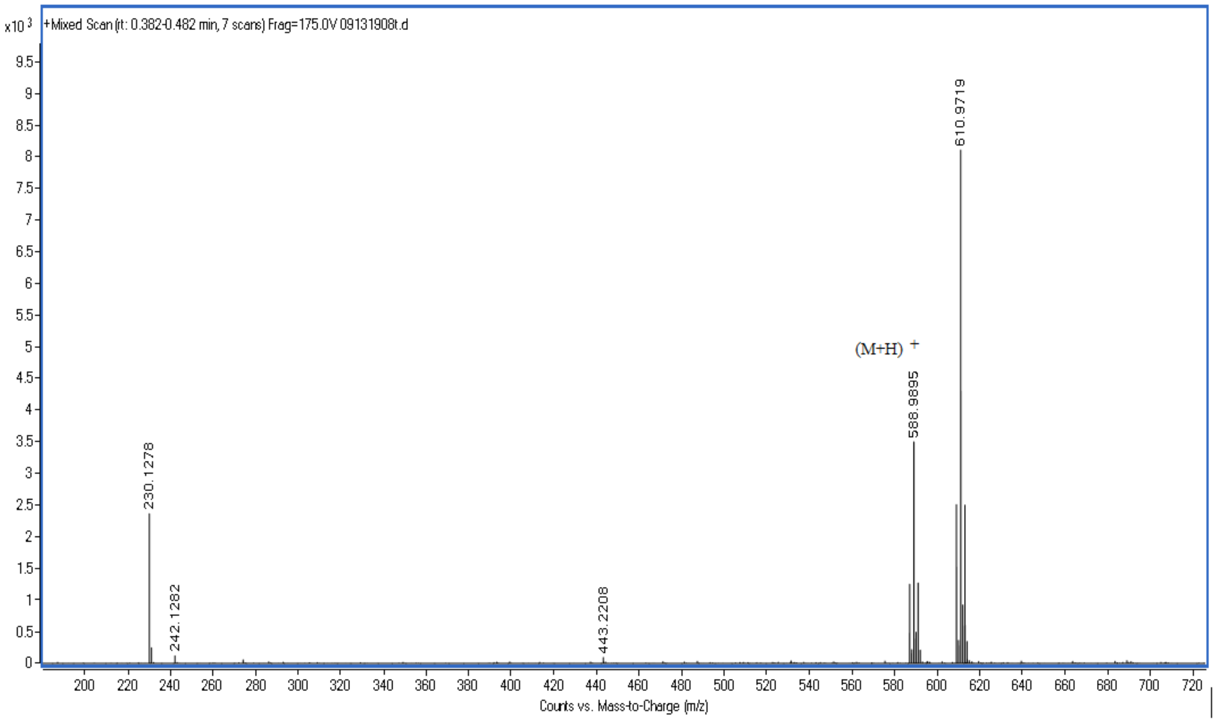

HRMS spectrum of compound **18b**

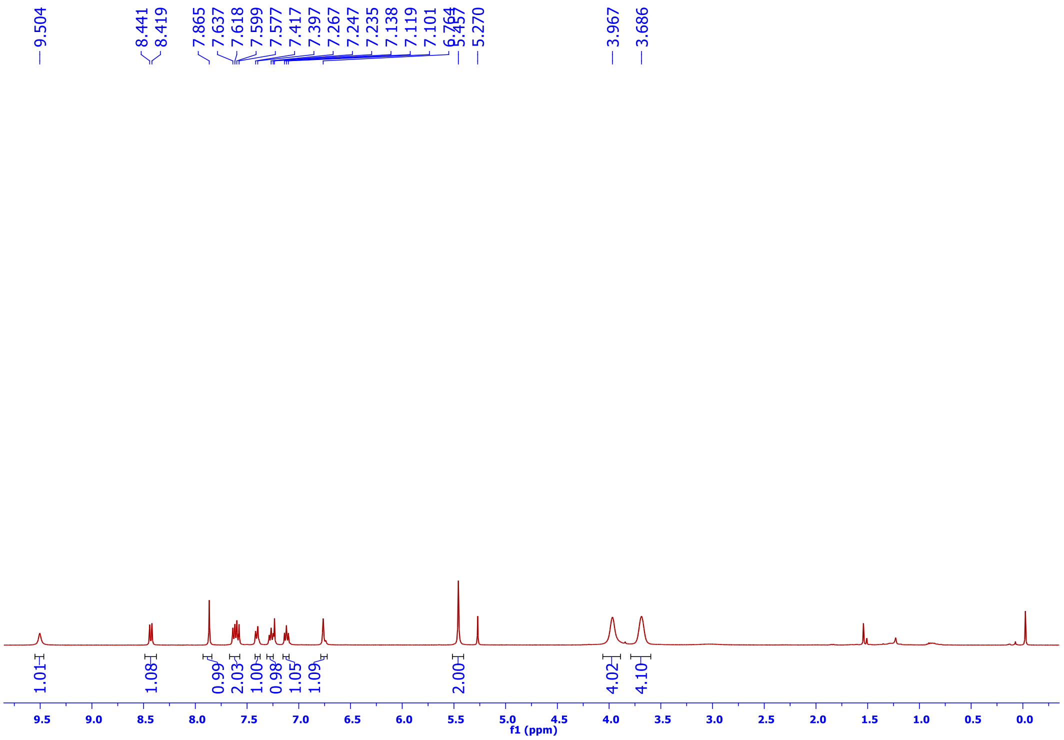

^{1}H NMR spectrum of compound **18c**

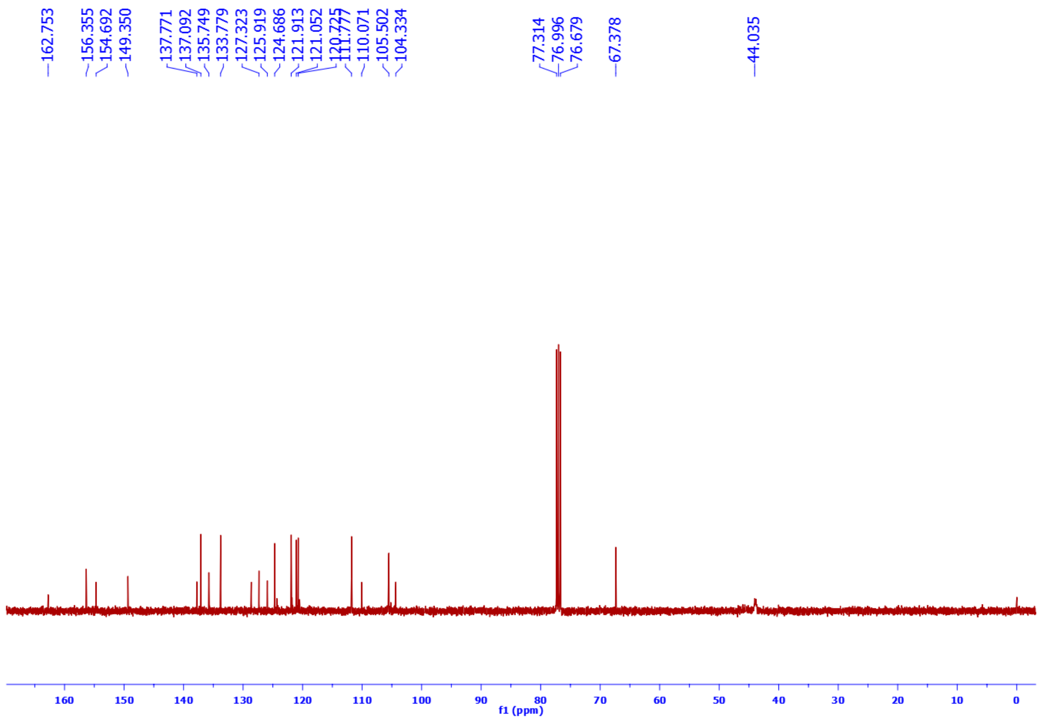

^{13}C NMR spectrum of compound **18c**

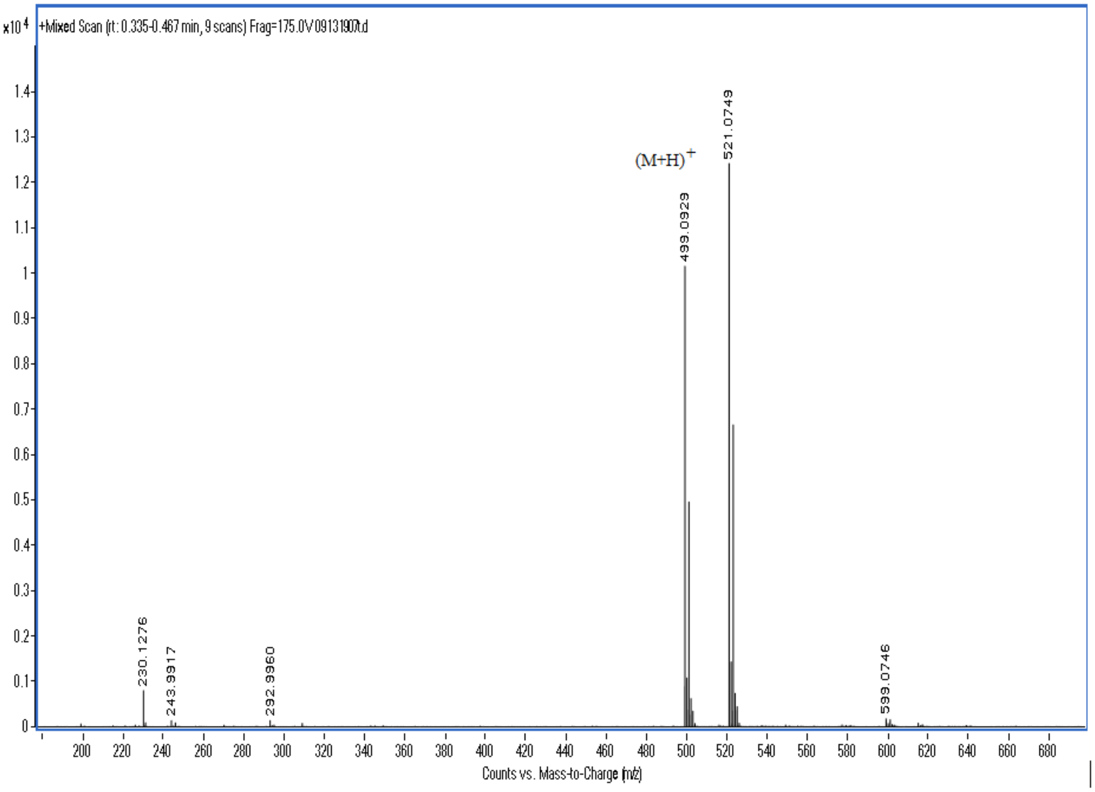

HRMS spectrum of compound **18c**

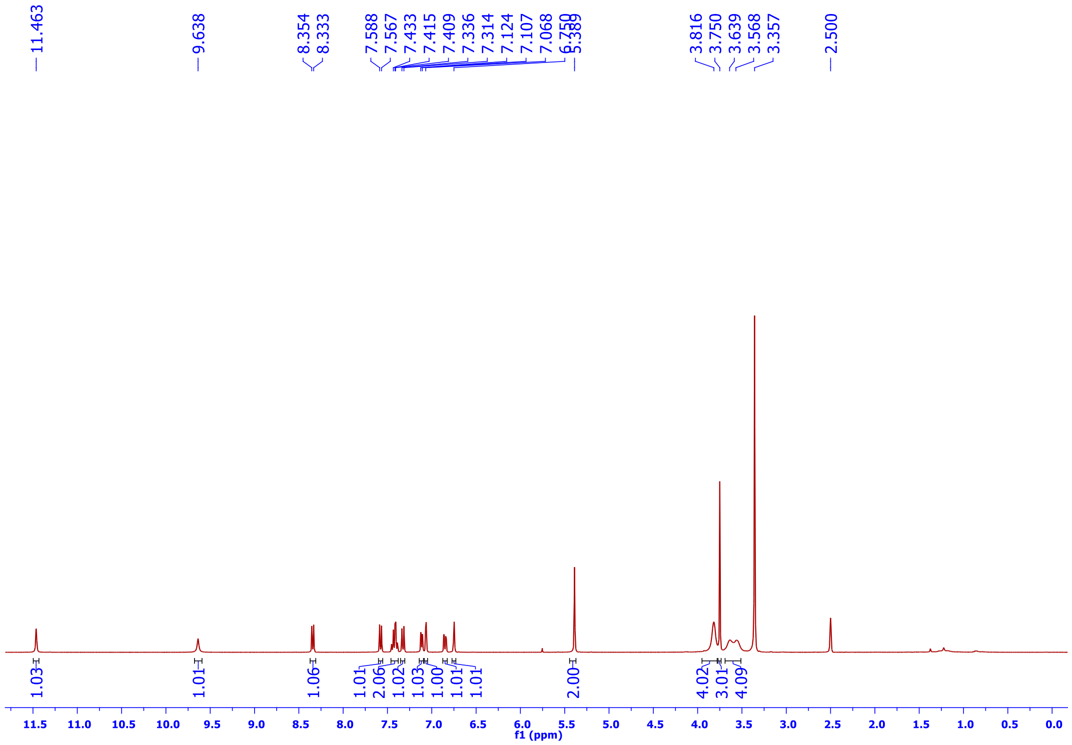

^{1}H NMR spectrum of compound **18d**

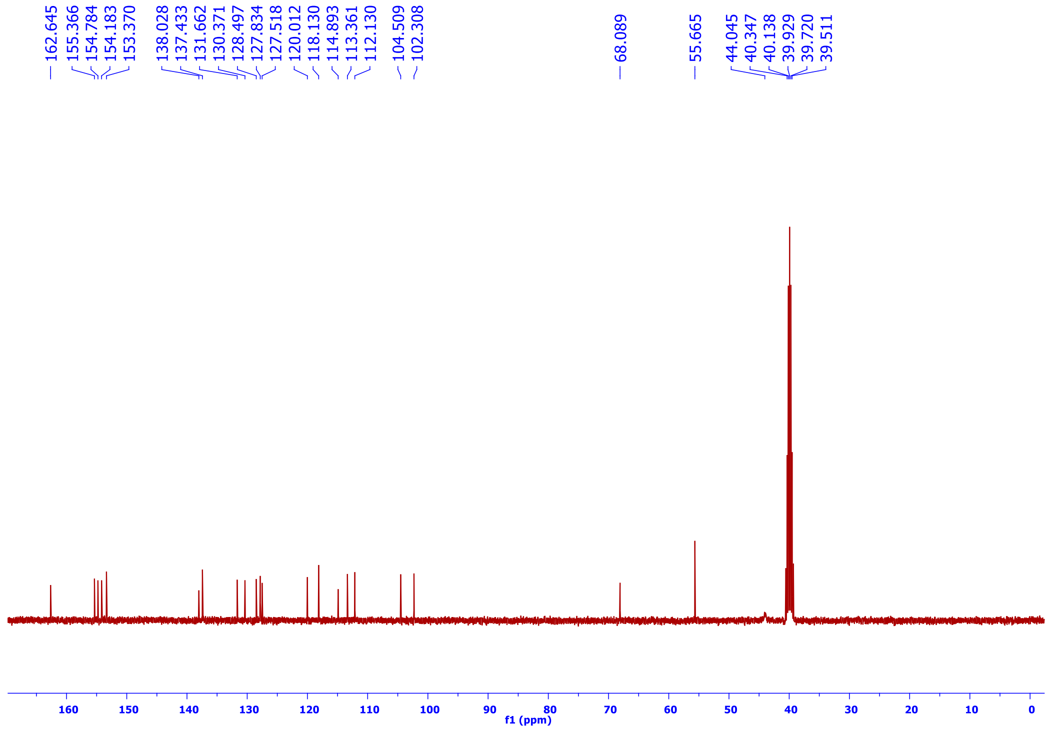

^{13}C NMR spectrum of compound **18d**

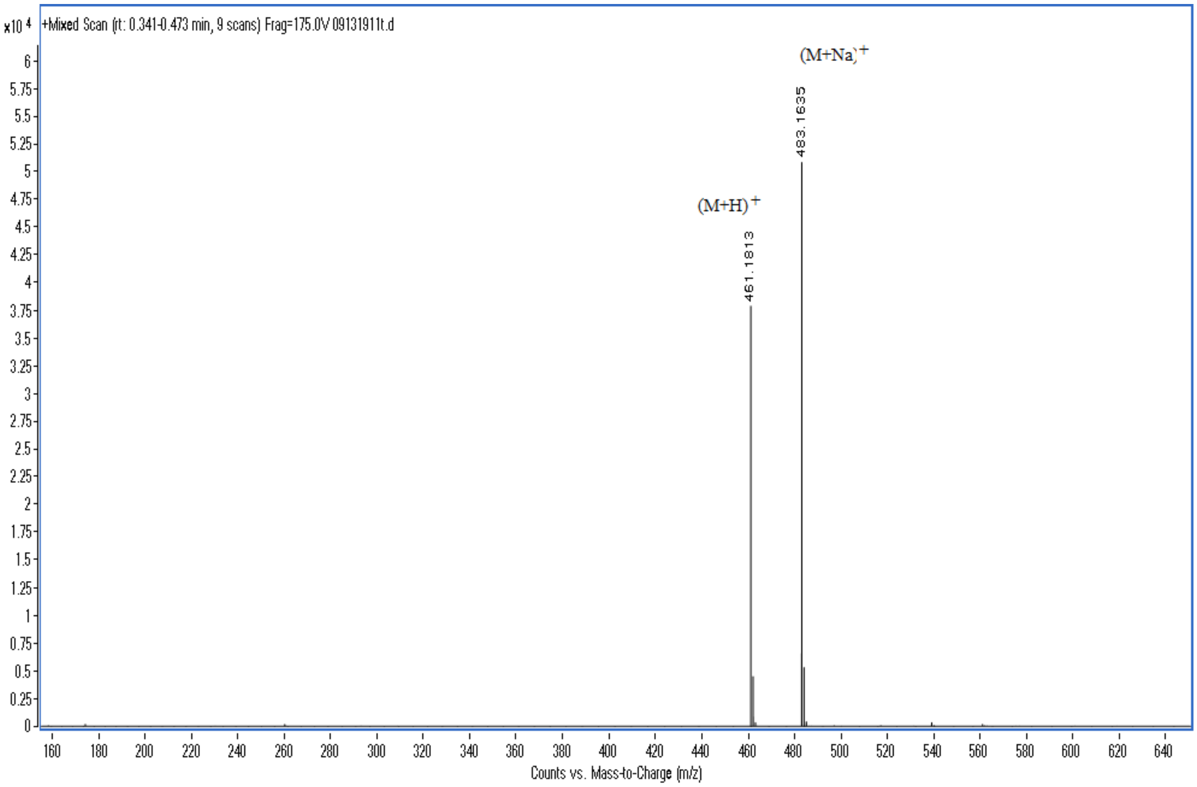

HRMS spectrum of compound **18d**

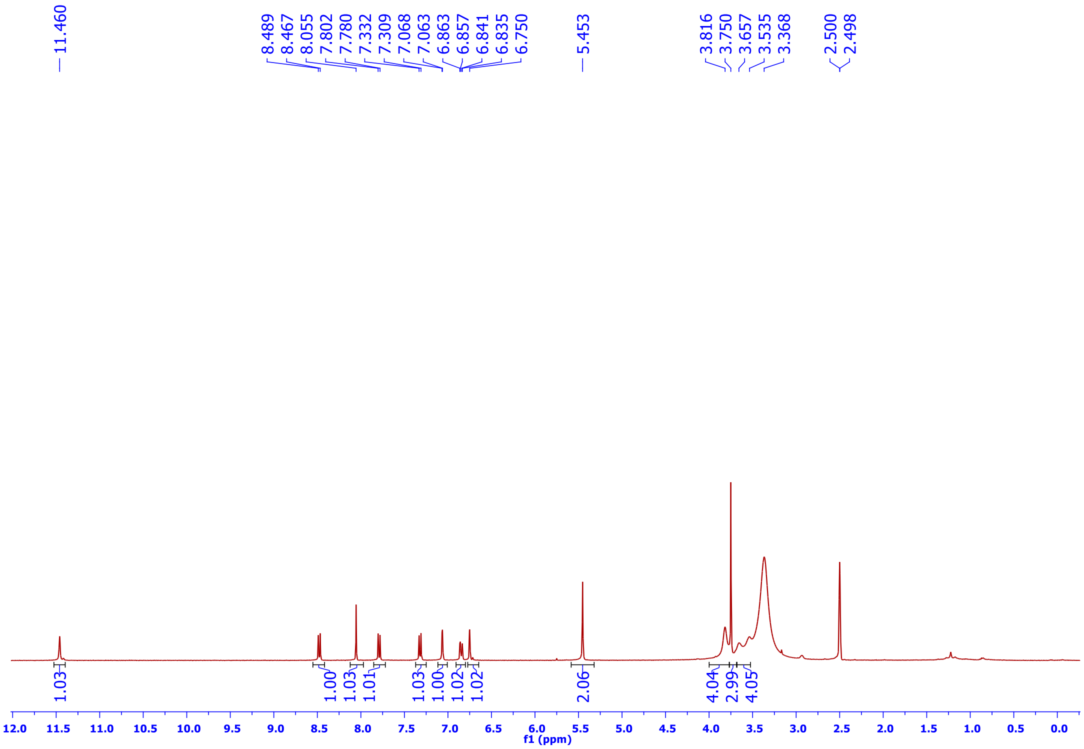

^{1}H NMR spectrum of compound **18e**

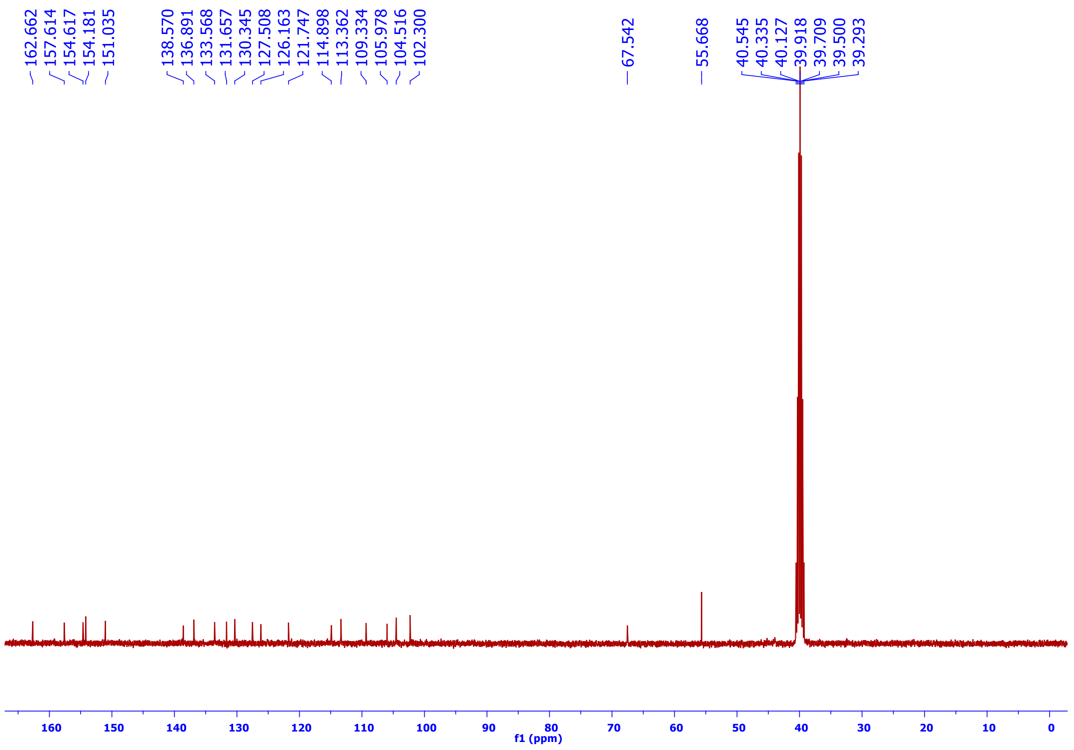

^{13}C NMR spectrum of compound **18e**

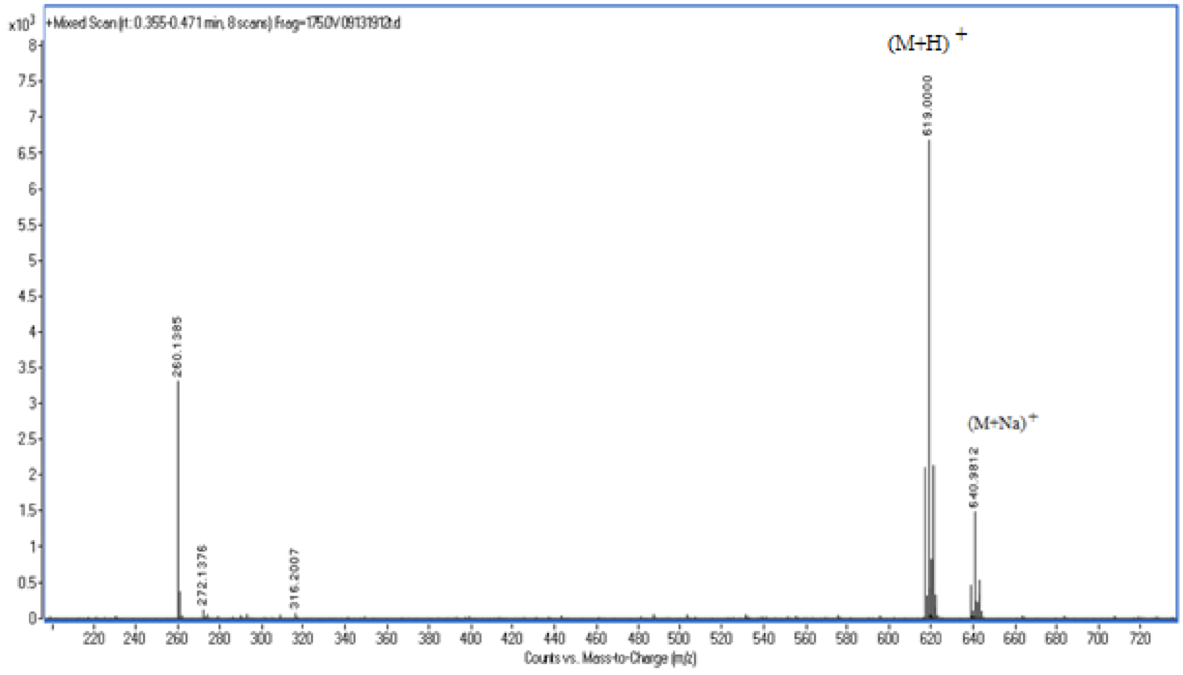

HRMS spectrum of compound **18e**

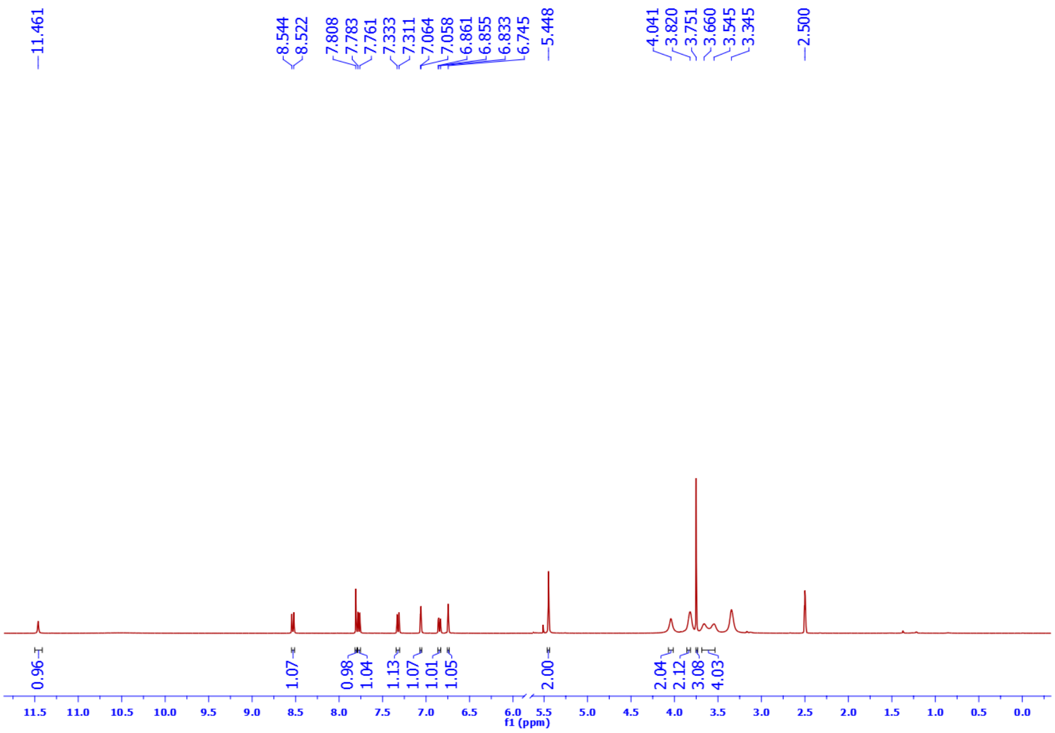

^{1}H NMR spectrum of compound **18f**

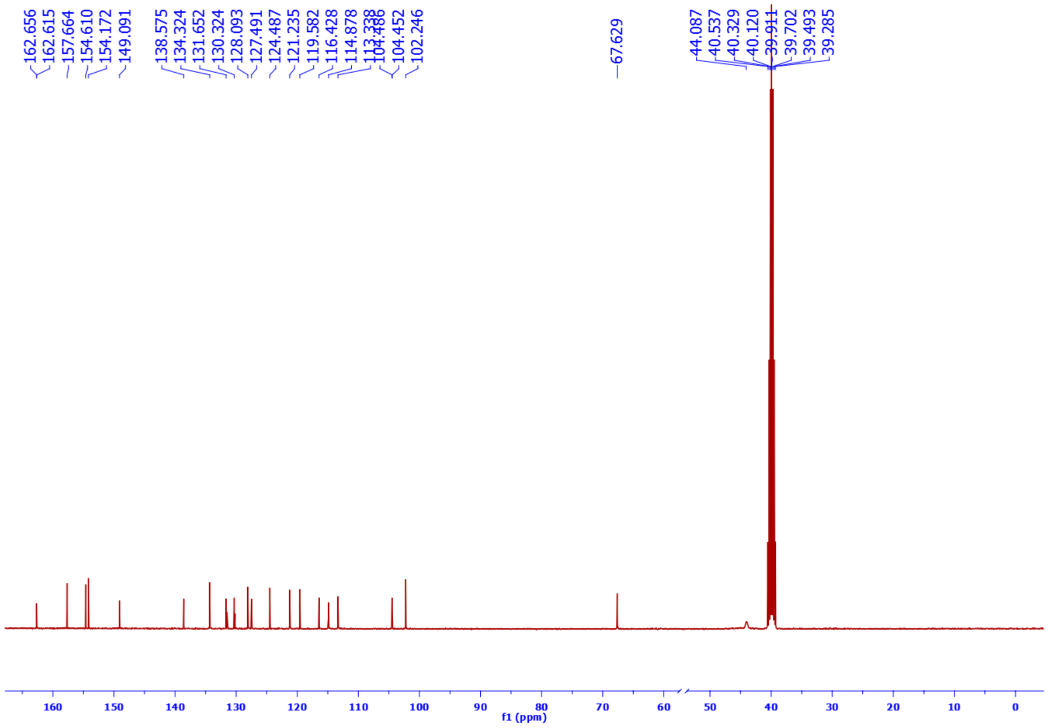

^{13}C NMR spectrum of compound **18f**

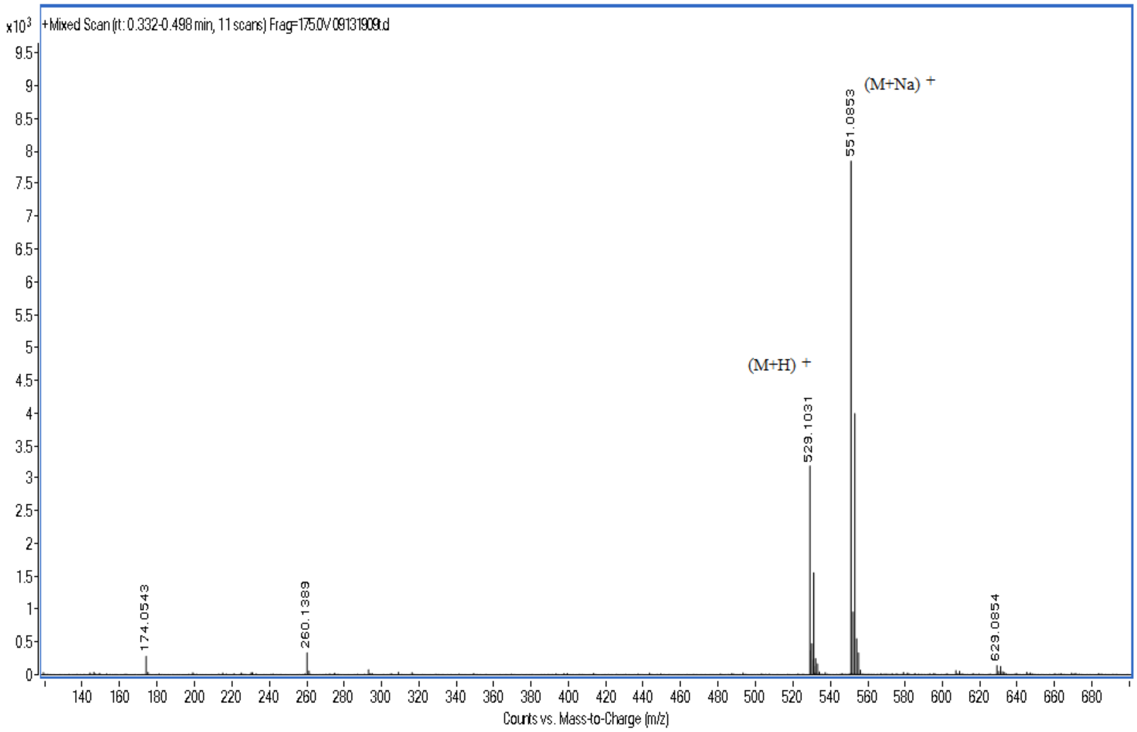

HRMS spectrum of compound **18f**

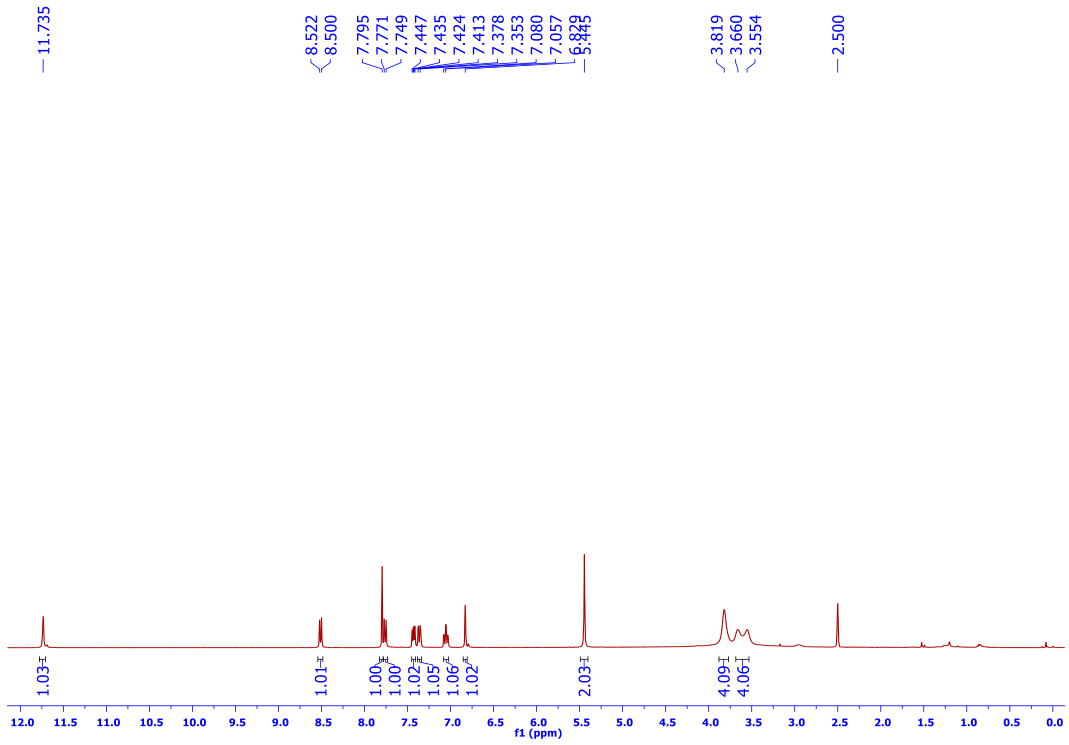

^{1}H NMR spectrum of compound **18g**

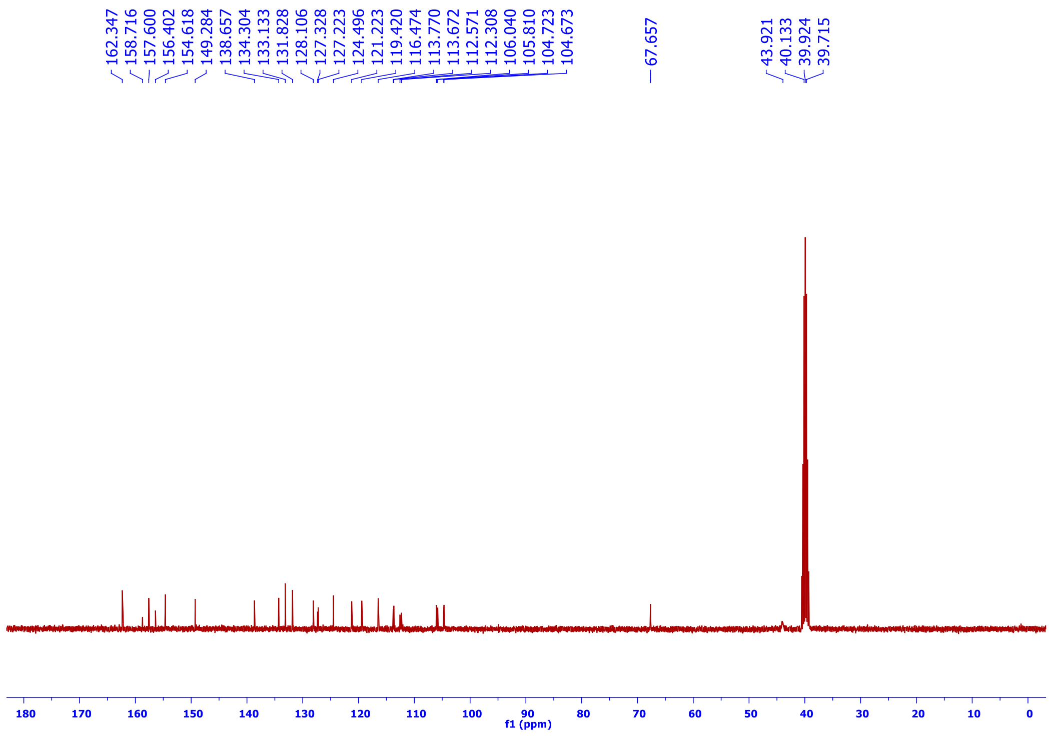

^{13}C NMR spectrum of compound **18g**

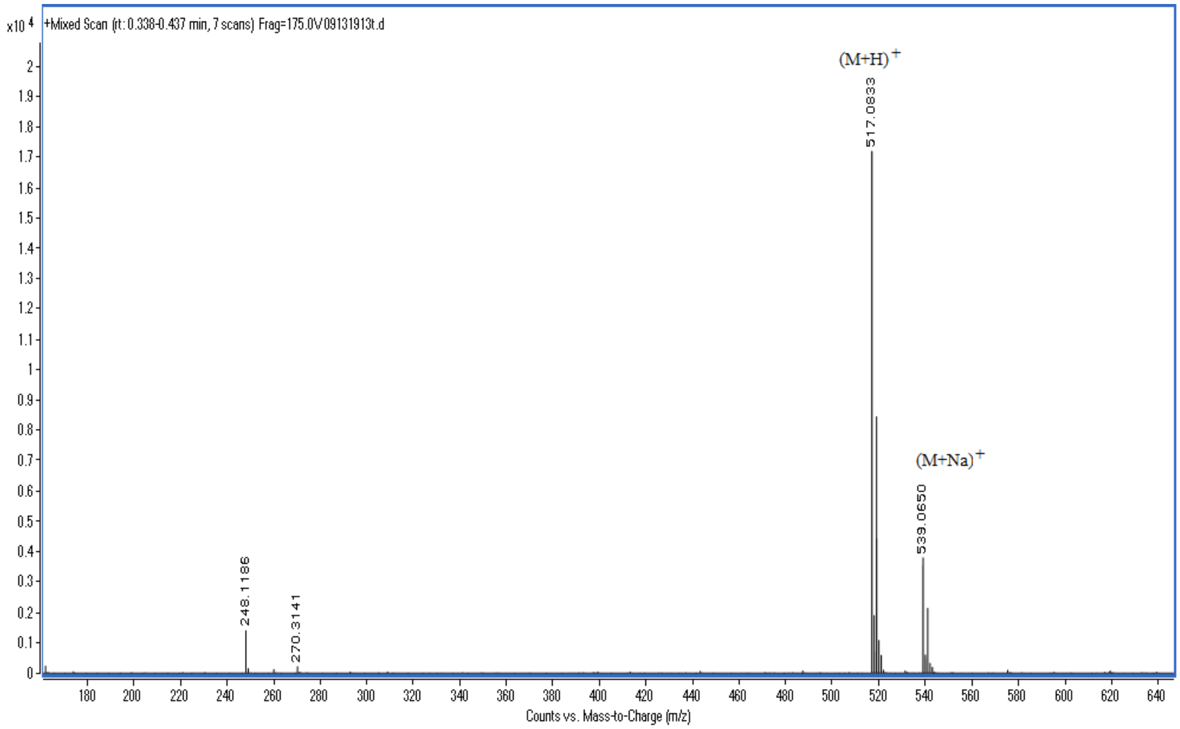

HRMS spectrum of compound **18g**

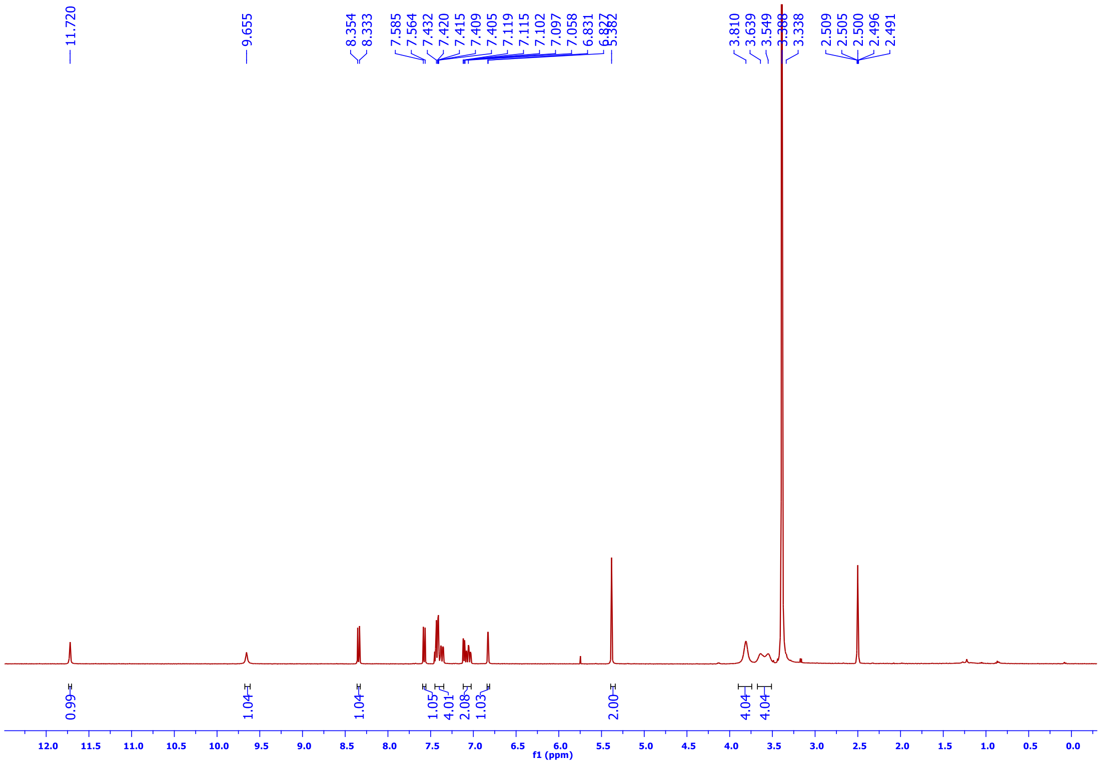

^{1}H NMR spectrum of compound **18h**

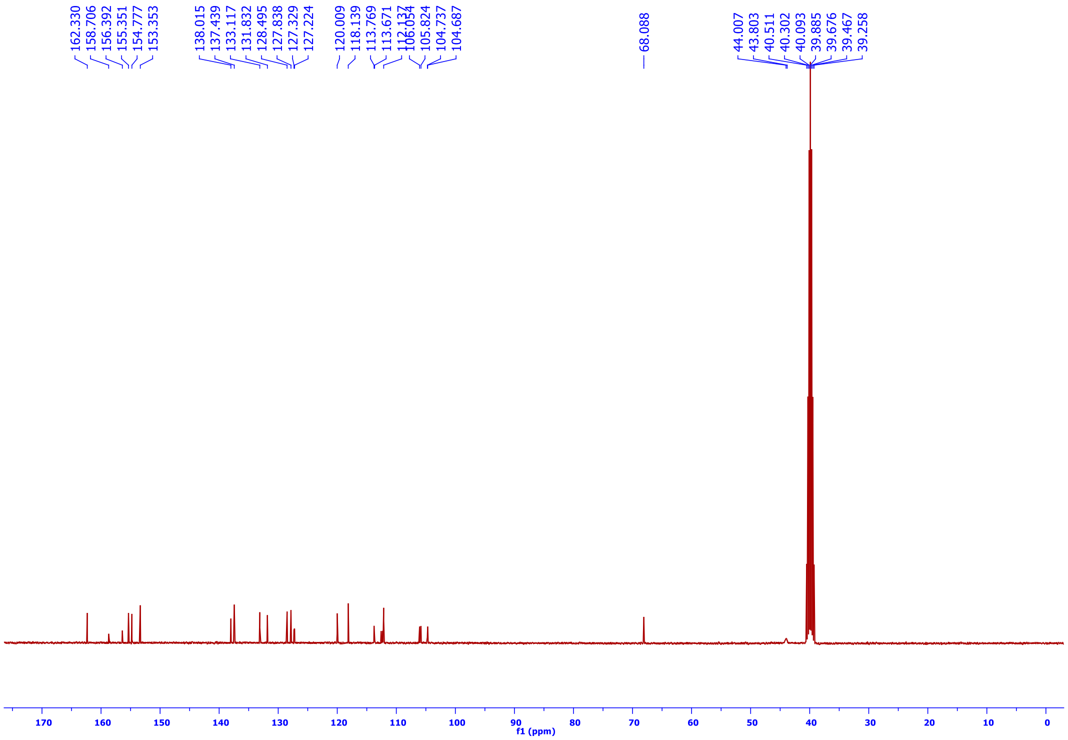

^{13}C NMR spectrum of compound **18h**

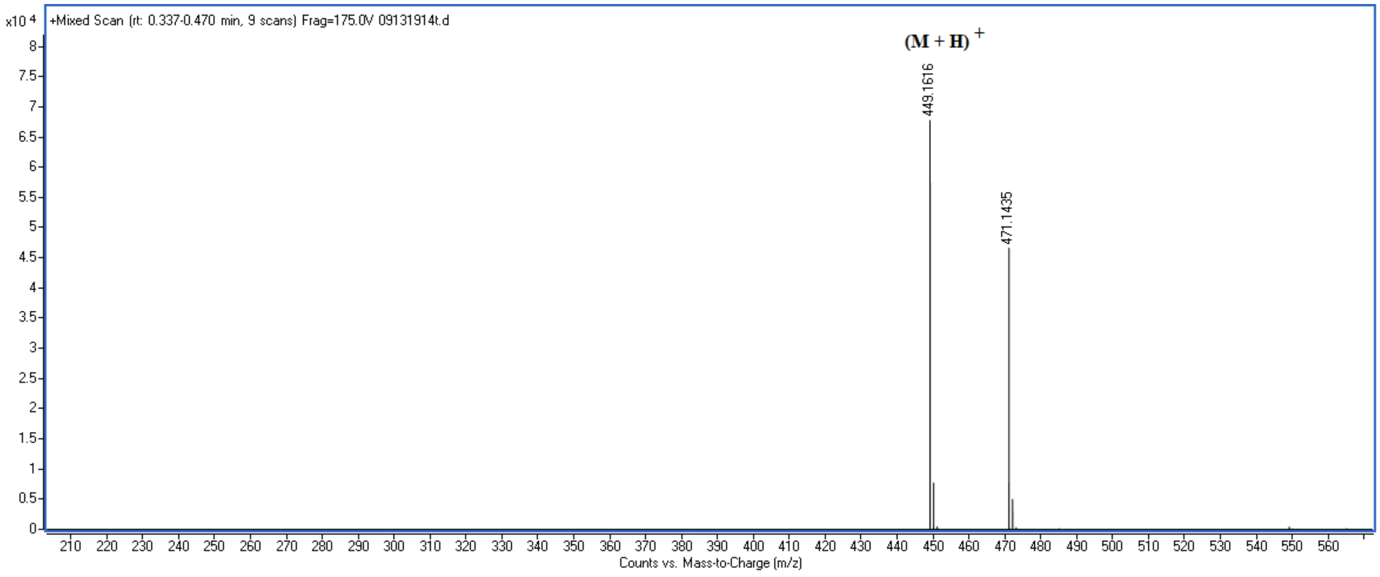

HRMS spectrum of compound **18h**
